# Stepwise phosphorylation of p65 promotes NF-κB activation and NK cell responses during target cell recognition

**DOI:** 10.1038/ncomms11686

**Published:** 2016-05-25

**Authors:** Hyung-Joon Kwon, Go-Eun Choi, Sangryeol Ryu, Soon Jae Kwon, Sun Chang Kim, Claire Booth, Kim E. Nichols, Hun Sik Kim

**Affiliations:** 1 Department of Biomedical Sciences, University of Ulsan College of Medicine, 86 Asanbyeongwon-Gil, Seoul 138-735, Korea; 2 Institute of Convergence Bio-Health, Dong-A University, Busan, Korea; 3 Department of Food and Animal Biotechnology, Department of Agricultural Biotechnology, Research Institute for Agriculture and Life Sciences, Seoul National University, Seoul 151-921, Korea; 4 Department of Biological Sciences, Korea Advanced Institute of Science and Technology, Daejeon 305-701, Korea; 5 Molecular Immunology Unit, Institute of Child Health, University College London, London WC1N 1EH, UK; 6 Department of Oncology, Division of Cancer Predisposition, St Jude Children's Research Hospital, Memphis, Tennessee 38105-3678, USA; 7 Department of Microbiology, University of Ulsan College of Medicine, Seoul 138-735, Korea; 8 Cellular Dysfunction Research Center, University of Ulsan College of Medicine, Seoul 138-735, Korea

## Abstract

NF-κB is a key transcription factor that dictates the outcome of diverse immune responses. How NF-κB is regulated by multiple activating receptors that are engaged during natural killer (NK)-target cell contact remains undefined. Here we show that sole engagement of NKG2D, 2B4 or DNAM-1 is insufficient for NF-κB activation. Rather, cooperation between these receptors is required at the level of Vav1 for synergistic NF-κB activation. Vav1-dependent synergistic signalling requires a separate PI3K-Akt signal, primarily mediated by NKG2D or DNAM-1, for optimal p65 phosphorylation and NF-κB activation. Vav1 controls downstream p65 phosphorylation and NF-κB activation. Synergistic signalling is defective in X-linked lymphoproliferative disease (XLP1) NK cells entailing 2B4 dysfunction and required for p65 phosphorylation by PI3K-Akt signal, suggesting stepwise signalling checkpoint for NF-κB activation. Thus, our study provides a framework explaining how signals from different activating receptors are coordinated to determine specificity and magnitude of NF-κB activation and NK cell responses.

## Abstract (web summary)

NK cell activation requires multiple signals. Here the authors show that while NKG2D, 2B4, or DNAM-1 receptor activation is insufficient to induce cytokine production, these signals synergize by Vav-1-mediated NF-κB multiphosphorylation, and this signaling checkpoint is defective in X-linked lymphoproliferative disease.

## Graphical abstract

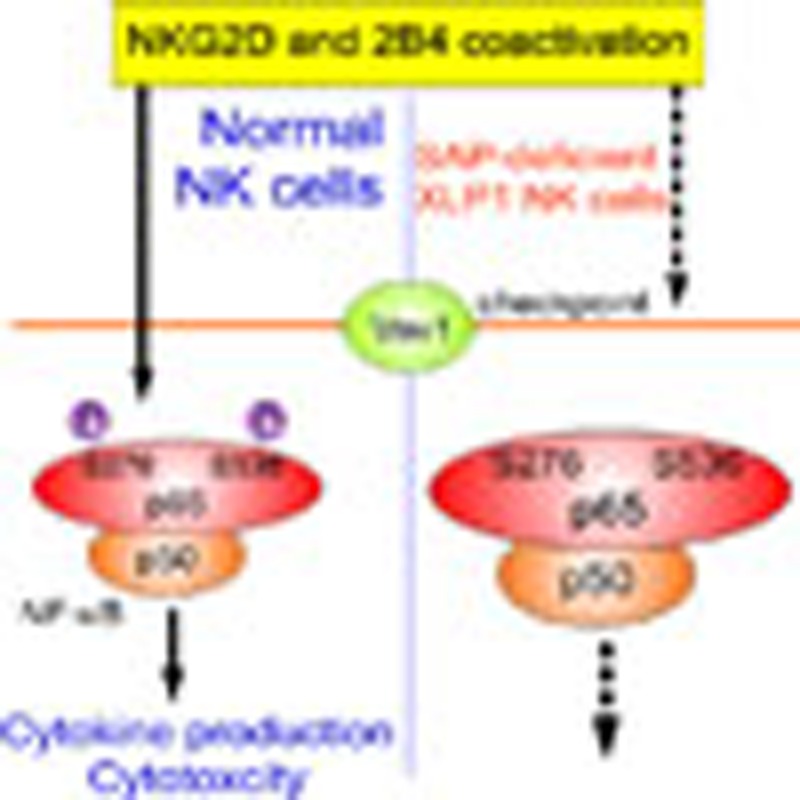

Natural killer (NK) cells serve pivotal roles in the early defence against transformed and virus-infected cells and also help shape adaptive immune responses by regulating antigen-presenting cells and T-cell responses^1^,^2^. These effector functions involve the secretion of cytokines such as Interferon-γ (IFN-γ) and tumor-necrosis factor-α (TNF-α) and the contact-dependent cytolysis of target cells^3^. NK cells can mount selective responses against diseased cells via integration of signals delivered by an array of germ line-encoded receptors^1^. To avoid inappropriate NK cell reactivity towards healthy cells, signals from multiple activating receptors are kept in check by inhibitory receptors such as killer cell Ig-like receptors and CD94-NKG2A heterodimer specific for MHC class I molecules on target cells. Even in the absence of such inhibition, engagement of a single activating receptor is generally insufficient to activate resting human NK cells because of a cell-intrinsic inhibition mechanism^4^. Efficient activation of resting NK cells requires combined stimulation by particular pairs of coactivation receptors, which function in combination (hereafter referred to as ‘synergistic' signalling). This differs from the activation of cytokine-stimulated NK cells, which no longer require coactivation^5^,^6^.

Receptor combinations that function synergistically include 2B4 (CD244) paired with NKG2D (CD314) or DNAM-1 (CD226), each with its unique signalling properties. 2B4 carries an ITSM motif in its cytoplasmic tail and transmits activation signals through recruitment of the small adaptor SAP and SAP-associated tyrosine kinase Fyn^7^,^8^. 2B4 signalling leads to Vav1, p38 MAPK, Erk and PLC-γ2 activation^9^. Notably, in NK cells from patients with the inherited immunodeficiency X-linked lymphoproliferative disease (XLP1), which lack functional SAP expression, 2B4 fails to activate and may instead deliver inhibitory signals^10^. NKG2D associates with the adaptor DAP10, which carries a YINM motif and signals through recruitment of phosphatidylinositol-3-kinase (PI3K) or Grb2-Vav1 complex^11^. NKG2D signalling involves Akt and MAPK Erk and Jnk. DNAM-1 signalling in NK cells remains unclear. DNAM-1 is associated with Fyn and phosphorylated by protein kinase C^12^, which is required for optimal differentiation of memory NK cells during cytomegalovirus infection^13^.

NK cell activation through receptors for ligands present on target cells can stimulate early cytokine and chemokine production, as well as target cell killing. A recent study on distinct NK subsets revealed CD56^dim^ NK cells, which are regarded as being specialized in cytotoxicity, to be a prominent source of cytokines upon contact with target cells^14^. Such cytokine responses, together with cytolytic activity, may constitute an important component of early immune surveillance. Although NK cell responses to soluble factors have been extensively studied (for example, IFN-γ production by interleukin (IL)-12 and IL-18) (ref. 15), the molecular mechanisms that control cytokine and chemokine production during NK-target cell contact remain largely undefined.

Signalling by various surface receptors modulates the activity of diverse transcription factors, which in turn induce the reprogramming of gene transcription for cytokine and chemokine production. A key transcription factor for such regulation is nuclear factor-κB (NF-κB)^16^,^17^. NK cells from patients deficient for NF-κB components, such as NF-kB essential modulator (NEMO) and inhibitor of κB (IκB) kinase β (IKKβ), demonstrate severe defects in IFN-γ production and cytotoxic function upon target cell recognition^18^,^19^, thus revealing the pivotal role of NF-κB in NK cell effector functions via receptor stimulation. The signalling pathways leading to NF-κB activation in NK cells have been characterized to some extent, but such studies are mostly confined to a few NK cell-activating receptors associated with immunoreceptor tyrosine-based activation motif (ITAM)-bearing adaptor molecules such as DAP12, FcRγ and CD3ζ (refs 20, 21). These include NKp30 in humans and NK1.1, Ly49D, Ly49H, CD16 and NKG2D in mice. Unlike human NKG2D, murine NKG2D can associate with both DAP12 and DAP10 (refs 22, 23). The signalling pathways downstream of ITAM-coupled receptors in NK cells are considered similar to those triggered by the antigen-specific receptors on B and T cells^9^. In contrast, it remains unclear how the signalling cascades induced by non-ITAM-associated receptors (for example, NKG2D, 2B4, DNAM-1) are coupled to NF-κB activation. Furthermore, because of multiple receptor–ligand interactions that occur during NK-target cell contact, it is important to understand how signals from different NK cell receptors are coordinated to control NF-κB activation.

Based on the requirement for cooperation among coactivation receptors to trigger effective cytokine production by resting NK cells, signalling by a single coactivation receptor may not suffice to activate NF-κB. Instead, it may require integration of disparate signals from coactivation receptors for proper NF-κB activation. In this study, we reveal that cooperative engagement of 2B4 with NKG2D or DNAM-1 is required to achieve signalling competence for full NF-κB activation. Furthermore, we uncover an unexpected checkpoint in NF-κB activation by revealing a requirement for complementary signals to converge at the level of Vav1 and downstream NF-κB p65 subunit. The pathophysiological relevance of this finding is supported by our identification of defective synergistic NF-κB activation and NK cell responses centred on Vav1 in XLP1 NK cells, which lack functional SAP, a signalling molecule required for 2B4-mediated signalling.

## Results

### Synergistic activation of NF-κB by NKG2D and 2B4

**Figure 1:**
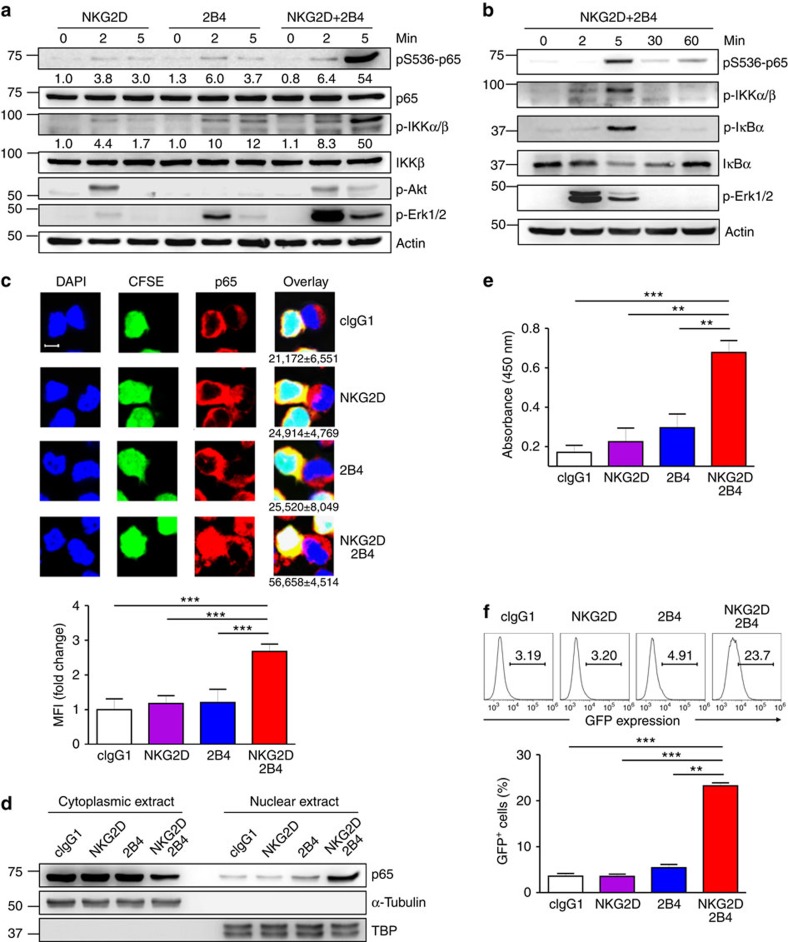
Synergistic activation of NF-κB by NKG2D and 2B4 co-engagement. (**a**) NKL cells rested in the absence of IL-2 for 24 h were stimulated with NKG2D and/or 2B4 by receptor crosslinking for the indicated time. Cell lysates were immunoblotted with Abs to phospho-p65 at serine 536 (pS536), p65, phospho-IKKα/β at serine 176/180 (pS176/180), IKKβ, phospho-Akt at serine 473 (pS473), phospho-Erk1 and 2, or actin. The normalized intensities of the phosphorylated p65 and IKKα/β relative to their total forms are presented. (**b**) Rested NKL cells were treated as in **a** to stimulate NKG2D and 2B4 for the indicated time. Lysates were immunoblotted for phospho-p65, phospho-IKKα/β, phospho-IκBα at serine 32/36 (pS32/36), IκBα, phospho-Erk1/2 or actin. (**c**) Representative confocal images (top) of conjugates between rested NKL cells loaded with CFSE (green) and P815 target cells as indicated. Conjugates were fixed, permeabilized and stained with 4,6-diamidino-2-phenylindole (DAPI; blue) and mAb to p65, anti-mouse IgG-Biotin followed with Alexa Fluor 647 (red)-Streptavidin. The number beneath the overlay image is the mean nuclear fluorescence intensity (MFI)±s.d. of p65 from ⩾50 NKL-target cell conjugates. Statistical bar charts (bottom) for MFI of p65 in the nucleus are represented as fold change. Values represent mean±s.d. Scale bar, 5 μm. (**d**) Rested NKL cells were stimulated with plate-immobilized mAbs to NKG2D and/or 2B4 for 1 h. Equal amounts of protein from cytoplasmic and nuclear extracts were immunoblotted with mAb to p65. (**e**) Nuclear extracts collected as in **d** were added into a 96-well plate immobilized with double-stranded oligonucleotide containing the consensus NF-κB-binding sequence. The amount of p65 bound to the oligonucleotide was measured by colorimetric assay. Values represent mean±s.d. (**f**) Rested NKL cells transduced with a κB-GFP reporter construct were stimulated with plate-immobilized mAbs to NKG2D and/or 2B4 for 6 h. GFP expression in NKL-κB-GFP cells was analysed by flow cytometry, and representative result (top) and statistical bar charts (bottom) are shown. Values represent mean±s.d. *P*<0.05; **P<0.01; ***P<0.001 (two-sided Student's *t*-test). Data are representative of at least three independent experiments.

Various stimulatory receptors trigger distinct signalling pathways that lead to NF-κB activation^24^. Such diverse NF-κB-activating stimuli, however, frequently converge on the IKK complex, and signalling cascade downstream of IKK appears to be well-conserved among most NF-κB activation pathways. This includes the phosphorylation and degradation of the IκB family, which releases bound NF-κB dimers for nuclear translocation and target gene transcription. So far, little is known about the mechanisms that couple non-ITAM-associated receptors to NF-κB activation during the triggering of natural cytotoxicity by NK cells. Thus, we first assessed NF-κB activation after stimulating NK cells via NKG2D, 2B4 or both. Stimulation of human NK cell line NKL with NKG2D or 2B4 alone resulted in detectable but weak phosphorylation of IKK and Erk, whereas co-engagement led to their synergistic phosphorylation (Fig. 1a). Such an increase was distinct from Akt phosphorylation, which is downstream of PI3K activation via NKG2D but not 2B4 (Fig. 1a). Likewise, phosphorylation of the downstream NF-κB p65 subunit (pS536-p65) resembled that of IKK. Such synergistic phosphorylation was evident at 5 min and decreased thereafter, which coincided with the phosphorylation and degradation of IκBα that links IKK activation and p65 nuclear translocation (Fig. 1b). We next performed microscopy experiments to assess nuclear translocation of p65 in individual NKL-target cell conjugates. To facilitate NKL cell identification, they were labelled with carboxyfluorescein succinimidyl ester (CFSE) and conjugated with unlabelled P815 target cells. In conjugated NKL cells, little or weak nuclear translocation of p65 was observed after stimulation with NKG2D or 2B4 alone (Fig. 1c). In contrast, significant nuclear translocation of p65 was detected in conjugated NKL cells following NKG2D and 2B4 co-engagement (Fig. 1c). Supporting this, analysis of subcellular fractions revealed a substantial increase in nuclear p65 and concomitant decrease in cytoplasmic p65 in NKL cells stimulated with NKG2D and 2B4 but neither receptor alone (Fig. 1d). Further, the amount of p65 bound to oligonucleotide containing consensus NF-κB-binding site was significantly increased in the nuclear extracts of NKL cells following NKG2D and 2B4 co-engagement (Fig. 1e). Finally, to evaluate the transcriptional activity of NF-κB, we generated NKL reporter (NKL-κB-GFP) cells that express GFP under the control of NF-κB transcription response elements. Proper functioning of the reporter cells was demonstrated by increased GFP expression following TNF-α treatment and its abrogation by prior treatment with BAY11-7082 that inhibits NF-κB activation (Supplementary Fig. 1). Compared with either receptor alone, NKG2D and 2B4 co-engagement induced an apparent and synergistic increase in the proportion of cells expressing GFP (Fig. 1f). Thus, via comprehensive analyses of conserved steps in the NF-κB activation pathway, we conclude that robust NF-κB activation is not achieved by stimulation with NKG2D or 2B4 alone, but instead relies on the integration of distinct signals from NKG2D and 2B4.

### NF-κB is required for NK cell functions by NKG2D and 2B4

Given NF-κB as a transcription factor important for gene regulation, we assessed gene expression after stimulating NK cells with NKG2D, 2B4 or both. Examination of a profile of 20 genes encompassing cytokines, chemokines, cytolytic pathway, death receptor pathway, NF-κB pathway, apoptosis, IL-2 receptor and cytotoxic granule exocytosis revealed a synergistic induction of diverse genes related to NK cell effector functions (Supplementary Fig. 2), correlating with the increase in NF-κB activation. Chemokine expression appears to be preferentially triggered by engagement of a single receptor (especially NKG2D), corroborating a recent study of the requirement for less stimulation to induce chemokine production compared with cytokine production or degranulation^14^. Nonetheless, NKG2D and 2B4 co-engagement still induced higher levels of chemokine and cytokine gene expression.

**Figure 2:**
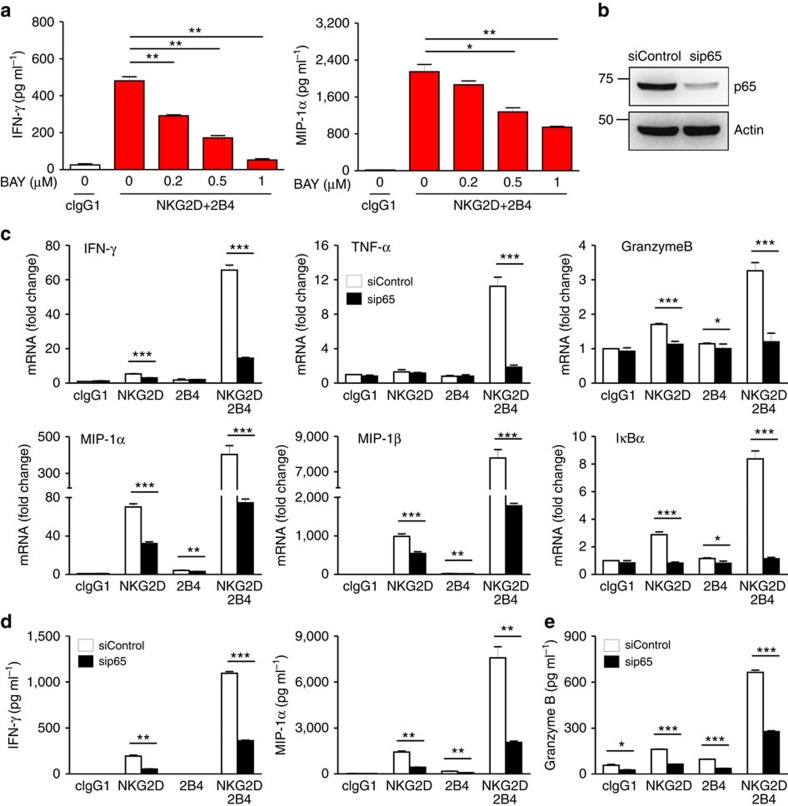
NF-κB is required for cytokine and chemokine gene expression by NKG2D and 2B4 coactivation. (**a**) Rested NKL cells were pretreated with a NF-κB inhibitor BAY11-7082 at the indicated dose for 1 h and then stimulated with both NKG2D and 2B4 for 8 h. Thereafter, IFN-γ and MIP-1α in the supernatants were measured by ELISA. Values represent mean±s.d. (**b**) NKL cells were transfected with 300 pmol of control siRNA or siRNA specific for p65. After 24 h, the cells were rested for another 24 h, and lysates were immunoblotted for p65 and actin. (**c**) Rested NKL cells transfected with control siRNA or p65-specific siRNA were stimulated with NKG2D and/or 2B4 for 3 h. Thereafter, total RNA was prepared from cells, reverse transcribed and the relative mRNA levels of IFN-γ, TNF-α, granzyme B, MIP-1α, MIP-1β and IκBα were determined by real-time PCR and normalized to β-actin mRNA. Values represent mean±s.d. (**d**) Rested NKL cells transfected with control siRNA or p65-specific siRNA were stimulated as in **c** for 8 h. IFN-γ and MIP-1α in the supernatants were measured by ELISA. Values represent mean±s.d. (**e**) Rested NKL cells that were transfected with control siRNA or p65-specific siRNA and stimulated with NKG2D and/or 2B4 for 2 h were used in a granzyme B release assay. Granzyme B in the supernatants was measured by ELISA. Error bars represent the s.d. *P<0.05; **P<0.01; ***P<0.001 (two-sided Student's *t*-test). Data are representative of at least three independent experiments.

Among the major genes induced by NF-κB are those encoding cytokines and chemokines. The synergy-dependent activation of NF-κB (Fig. 1) and mRNA expression of IFN-γ and MIP-1α (Supplementary Fig. 2) prompted us to test whether NF-κB is required for NK cells to produce cytokines and chemokines following NKG2D and 2B4 engagement. The synergistic production of IFN-γ and MIP-1α by NKL cells was diminished in a dose-dependent manner following BAY11-7082 treatment (Fig. 2a). To directly probe the role of NF-κB, we silenced the expression of NF-κB p65 subunit, using small interfering RNA (siRNA) (Fig. 2b). p65 knockdown caused marked reductions in mRNA expression of IFN-γ, TNF-α, MIP-1α/β, granzyme B and IκBα induced by NKG2D and 2B4 co-engagement (Fig. 2c), confirming the dependence of their transcription on NF-κB. Similarly, the synergistic production of IFN-γ and MIP-1α was significantly reduced by p65 knockdown (Fig. 2d). To confirm this finding in the context of physiological receptor–ligand interactions, we stimulated NKL cells with P815 cells expressing ULBP1 (a ligand for NKG2D) and/or CD48 (a ligand for 2B4). Consistently, synergistic production of IFN-γ and MIP-1α following physiological stimulation was significantly diminished by p65 knockdown (Supplementary Fig. 3).

Given the reports showing defective cytotoxicity of NK cells from patients with NF-κB deficiency^18^,^19^, we next assessed whether NF-κB is also required for cytotoxic degranulation of NK cells. The synergistic increase in degranulation, as assessed by granzyme B release, was significantly decreased by p65 knockdown (Fig. 2e). Collectively, NF-κB could play an indispensable role in the production of cytokines and chemokines, as well as the release of granzyme B by NK cells via non-ITAM-associated receptors NKG2D and 2B4.

We next determined whether the findings obtained from NKL cells were applicable to primary NK cells. NF-κB activation was assessed using purified NK cells on a per-cell basis by flow cytometry-based analysis of p65 phosphorylation. Similar to experiments using NKL cells (Supplementary Fig. 4), the proportion of responding cells synergistically increased following combined stimulation of primary NK cells with NKG2D and 2B4 (Supplementary Fig. 5a). NKG2D and 2B4 co-engagement also led to synergistic increase in the proportion of NK cells expressing IFN-γ or TNF-α (Supplementary Fig. 5b), which was diminished after BAY11-7082 treatment (Supplementary Fig. 5c). BAY11-7082 did not significantly affect the viability of NKL cells and primary NK cells, as assessed by annexin-V/PI staining (Supplementary Fig. 6). Collectively, these results suggest that NKG2D and 2B4 coactivation is required to overcome a threshold for NF-κB activation, leading to synergistic cytokine production by NK cells.

### Disparate signals converge on the NF-κB p65 subunit

**Figure 3:**
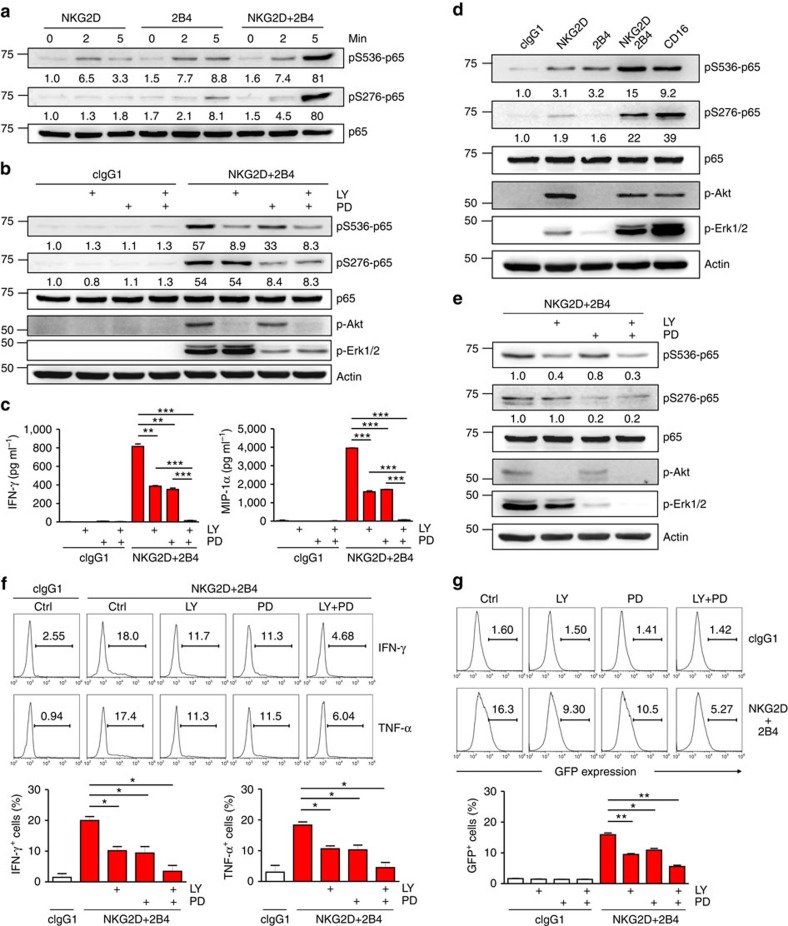
NKG2D and 2B4 coactivation induces disparate and cooperative signals for NF-κB activation. (**a**) Rested NKL cells were stimulated with NKG2D and/or 2B4 by receptor crosslinking for the indicated time. Cell lysates were immunoblotted for phospho-p65 at serine 536 (pS536), phospho-p65 at serine 276 (pS276) or p65. The normalized intensities of the phosphorylated p65 relative to total p65 are presented. (**b**) Rested NKL cells were stimulated with NKG2D and 2B4 for 5 min after pretreatment with PI3K inhibitor (LY294002; 20 μM) and/or MEK inhibitor (PD98059; 20 μM) for 30 min. Lysates were analysed by immunoblotting for the indicated phosphorylations of p65. (**c**) Cytokine release assays with rested NKL cells after pretreatment with 20 μM LY294002 and/or 20 μM PD98059 for 30 min and then stimulation with NKG2D and 2B4 for 12 h in the presence of the inhibitor. Thereafter, IFN-γ (left) and MIP-1α (right) in the supernatants were measured by ELISA. Values represent mean±s.d. (**d**) Primary rested NK cells after expansion were stimulated with the indicated receptors by receptor crosslinking for 5 min. Lysates were analysed by immunoblotting as in **b**. (**e**) Primary rested NK cells after expansion were stimulated and analysed by immunoblotting as in **b**. (**f**) Frequency of NK cells that displayed IFN-γ or TNF-α expression after pretreatment of PBMCs with 20 μM LY294002 and/or 20 μM PD98059 for 1 h and then stimulation with P815 target cells as indicated in the presence of the inhibitor. After incubation for 6 h, cells were stained and analysed by flow cytometry. Representative result (top) and statistical bar charts (bottom) from three experiments are shown. Values represent mean±s.e.m. (**g**) Rested NKL-κB-GFP cells were pretreated with 20 μM LY294002 and/or 20 μM PD98059 for 1 h and then stimulated with plate-immobilized NKG2D and 2B4 for 6 h. GFP expression in the reporter NKL cells was analysed by flow cytometry, and representative result (top) and statistical bar charts (bottom) are shown. Values represent mean±s.d. *P<0.05; **P<0.01; ***P<0.001 (two-sided Student's *t*-test). Data are representative of at least three independent experiments.

NF-κB activation occurs primarily via IKK-dependent phosphorylation and degradation of IκB proteins, followed by nuclear translocation of the released NF-κB dimers. In addition, optimal NF-κB activation relies on post-translational modifications of NF-κB subunits, such as p65 phosphorylation, by specific protein kinases^25^,^26^. These emerging and additional layers of NF-κB regulation represent an important means for crosstalk between different signalling pathways and determining context-specific transcriptional responses^27^. NKG2D and 2B4 co-engagement induces two distinct pathways that lead to the activation of PI3K-Akt and Vav1-dependent synergistic signalling involving PLC-γ2 and Erk^4^. Given the site-selective phosphorylation of p65 through Akt and Erk pathways^26^, Vav1-dependent synergy achieved by a proximal convergence of signals from coactivation receptors may not suffice to activate NF-κB. Although stimulation of NKL cells with NKG2D or 2B4 alone induced weak p65 phosphorylation at serine 276 and 536, NKG2D and 2B4 co-engagement functioned synergistically to increase their phosphorylation (Fig. 3a). In support, synergistic phosphorylation of p65 was observed after stimulation with P815 cells expressing ULBP1 and CD48 (Supplementary Fig. 7). To assess the dependence of such phosphorylation on Akt and Erk pathways, NKL cells were treated with an inhibitor of PI3K (LY294002) or MEK (PD98059), which block Akt or Erk activation, respectively. Notably, p65 phosphorylation at serine 276 fully depended on NKG2D and 2B4 co-engagement-induced Erk activation (Fig. 3b). In contrast, p65 phosphorylation at serine 536 largely depended on NKG2D-induced PI3K-Akt. The PI3K-Akt and synergistic Erk pathway appear to be mutually independent, given the insensitivity of synergistic Erk activation to PI3K inhibitors and Akt activation to MEK inhibitors^4^. The functional significance of these pathways was supported by significant impairment of IFN-γ and MIP-1α production by an inhibitor of each pathway and further by the combined inhibition of both pathways (Fig. 3c). To probe the direct role of these kinase pathways, we performed knockdown of Akt1 and/or Erk2 in NKL cells. Both Akt1 and Erk2 were required for optimal p65 phosphorylation and IFN-γ and MIP-1α production following coactivation, complementing the results with small-molecule inhibitors (Supplementary Fig. 8).

We next assessed these findings using primary rested human NK cells. Similar to the results seen in NKL cells, NKG2D and 2B4 co-engagement led to synergistic phosphorylation of p65 at serine 276 and 536 (Fig. 3d). Moreover, we observed that phosphorylation of S276-p65 was selectively dependent on Erk pathway, whereas phosphorylation of S536-p65 largely relied on Akt(Fig. 3e). As expected, synergistic expression of IFN-γ and TNF-α was diminished by inhibiting Akt, Erk or both pathways (Fig. 3f), correlating with reduced NF-κB activation, as determined using reporter cells (NKL-κB-GFP; Fig. 3g). The combined inhibition of Akt and Erk pathways demonstrated an additive effect on dampening NF-κB activation, suggesting separate involvement of Akt and Erk pathways in NF-κB activation.

To determine whether p65 phosphorylation is similarly induced by a different combination of coactivation receptors, primary NK cells were examined after engaging DNAM-1, 2B4 or both. DNAM-1 synergizes with 2B4 to trigger effector functions^28^. Notably, crosslinking DNAM-1 induced Akt phosphorylation, as did NKG2D, and 2B4 co-engagement induced a synergistic phosphorylation of Erk, an apparent dual phosphorylation of p65, and synergistic NF-κB activation (Supplementary Fig. 9a,b). These suggest that the basis for NF-κB activation is shared between DNAM-1 and NKG2D. In contrast, crosslinking CD16, which mediates antibody-dependent cellular cytotoxicity and is sufficient for cytokine production, induced dual phosphorylation of p65 in addition to Akt and Erk phosphorylation (Fig. 3d). These suggest distinct regulation of p65 phosphorylation by coactivation receptors for natural cytotoxicity that are incapable of activating alone. Thus, co-engagement of 2B4 with NKG2D, or DNAM-1, was required to achieve signalling competence for p65 phosphorylation and NF-κB activation.

### Signal amplification is insufficient for NF-κB activation

**Figure 4:**
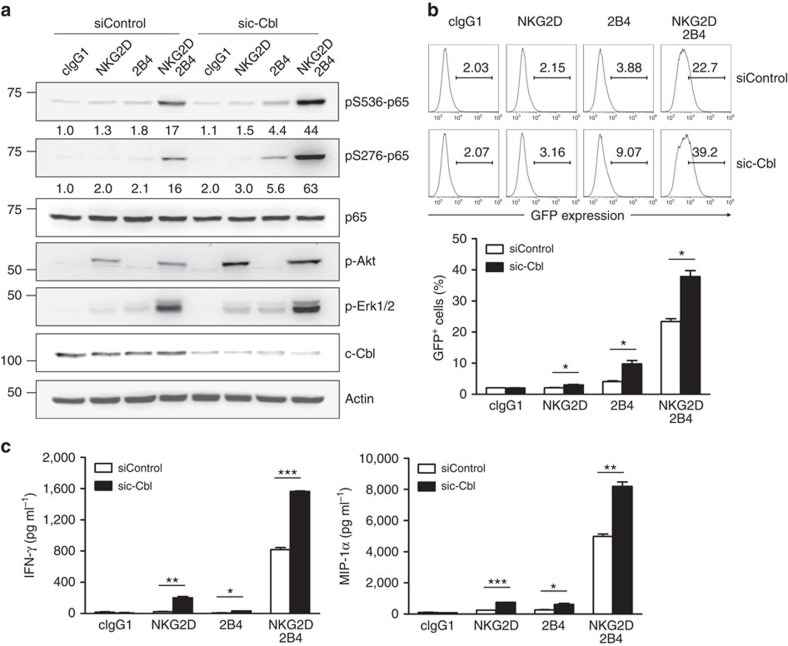
c-Cbl depletion augments but is not sufficient for NF-κB activation. (**a**) Rested NKL cells transfected with control siRNA or c-Cbl-specific siRNA were stimulated through the indicated receptors for 5 min. Cell lysates were immunoblotted for phospho-p65 at serine 536 (pS536), phospho-p65 at serine 276 (pS276), p65, phospho-Akt at serine 473 (pS473), phospho-Erk1 and 2, c-Cbl or actin The normalized intensities of the phosphorylated p65 relative to p65 are presented. (**b**) Rested NKL-κB-GFP cells transfected with control siRNA or c-Cbl-specific siRNA were stimulated with plate-immobilized mAbs specific for NKG2D and/or 2B4 for 6 h. GFP expression in NKL-κB-GFP cells was analysed by flow cytometry, and representative result (top) and statistical bar charts (down) are shown. Values represent mean±s.d. (**c**) Cytokine release assays with rested NKL cells transfected with control siRNA or c-Cbl-specific siRNA and stimulated with NKG2D and/or 2B4 for 8 h. IFN-γ and MIP-1α in the supernatants were measured by ELISA. Values represent mean±s.d. *P<0.05; **P<0.01; ***P<0.001 (two-sided Student's *t*-test). Data are representative of at least three independent experiments.

The dependence of NK cell activation on synergistic signals is, in part, relieved after c-Cbl knockdown^4^. Thus, we examined whether depleting c-Cbl enables NKG2D or 2B4 to bypass the requirement for synergistic signals to induce p65 phosphorylation and NF-κB activation. c-Cbl knockdown slightly enhanced p65 phosphorylation at serine 536 and 276 in response to NKG2D or 2B4 alone (Fig. 4a). However, it remained lower than p65 phosphorylation by their synergistic coactivation. In contrast, c-Cbl knockdown substantially enhanced p65 phosphorylation at both serines following synergistic coactivation.

Given the role of Akt and Erk in the phosphorylation of S536-p65 and S276-p65, we next assessed the effects of c-Cbl knockdown on Akt and Erk phosphorylation. Although Akt phosphorylation induced by NKG2D was markedly augmented by c-Cbl knockdown, it was undetectable following 2B4 stimulation even after c-Cbl knockdown (Fig. 4a). In comparison, c-Cbl knockdown caused a small increase in Erk phosphorylation by NKG2D or 2B4 alone, but an apparent increase by their synergistic coactivation. These results suggest that c-Cbl knockdown may amplify signal input, but not trigger disparate signals for NF-κB activation.

The extent of pS276-p65 induced by NKG2D, 2B4 or both correlated with the level of Erk phosphorylation (Fig. 4a). In contrast, the correlation between pS536-p65 and Akt phosphorylation was observed following NKG2D and 2B4 coactivation, but not NKG2D alone, although there was comparable Akt phosphorylation. This suggests a checkpoint that restrains pS536-p65 by Akt pathway, which could be overcome by synergistic coactivation. In support, NF-κB activation and production of IFN-γ and MIP-1α by NKG2D and 2B4 coactivation but not by a single receptor was markedly enhanced after c-Cbl knockdown (Fig. 4b,c). Together, signal amplification from individual receptor could not compensate for the lack of complementary signals from its partner receptor for NF-κB activation.

### Defective NF-κB activation in XLP1 NK cells by NKG2D and 2B4

We next assessed whether NF-κB activation requires cooperation between coactivation receptors in pathophysiological contexts. XLP1 is characterized by severe immunodeficiency resulting from mutations in the *SH2D1A* gene encoding the SAP protein^29^,^30^,^31^. Given the requirement of SAP for 2B4-dependent NK cell activation^32^,^33^, we hypothesized that NK cells from XLP1 patients would demonstrate defects in NF-κB activation and NK cell responses following coactivation. We first tested the requirement of SAP for NK cell activation during NKG2D and 2B4 synergy by performing siRNA-mediated knockdown of SAP in NKL cells (Supplementary Fig. 10a). The synergistic increases in Ca^2+^ mobilization, NF-κB activation and IFN-γ and MIP-1α release were markedly diminished by SAP knockdown (Supplementary Fig. 10b,c,d). Likewise, SAP knockdown markedly reduced the synergistic increase in p65 phosphorylation and the proportion of NK cells expressing IFN-γ in primary NK cells following NKG2D and 2B4 coactivation (Supplementary Fig. 11). The small increase in IFN-γ expression by ligating 2B4 but not NKG2D alone was also selectively decreased by SAP knockdown.

**Figure 5:**
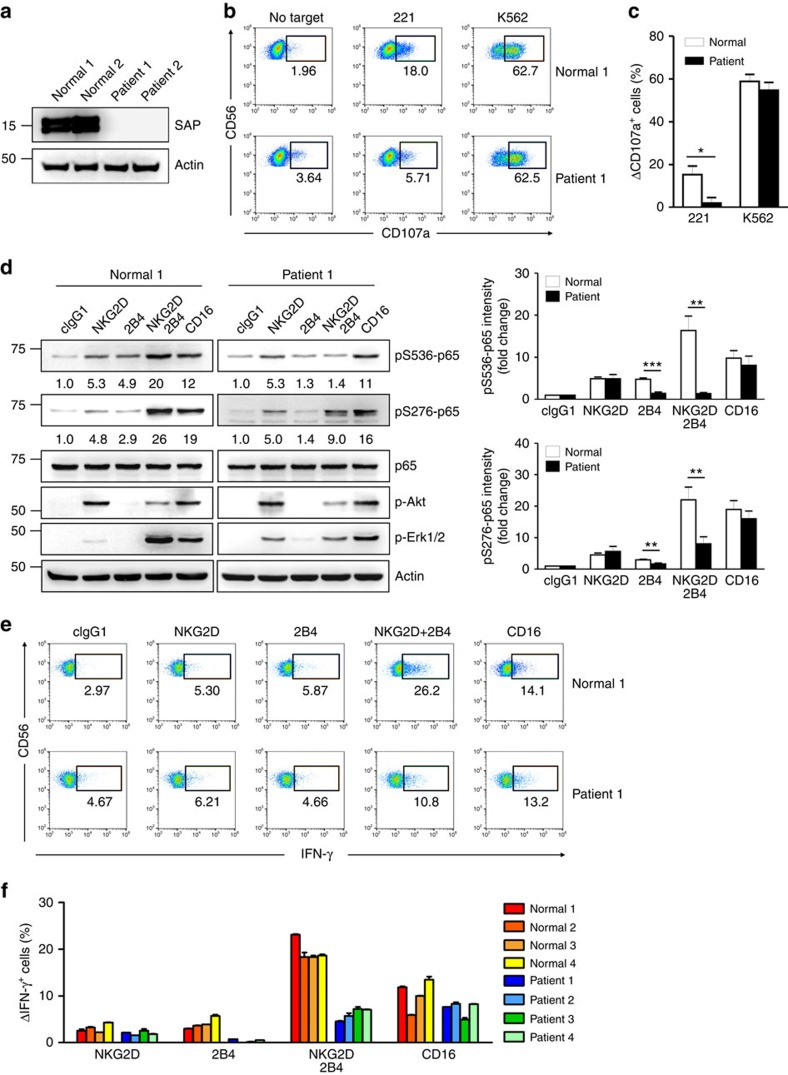
XLP1 NK cells have defects in NF-κB activation and IFN-γ expression in response to coactivation by NKG2D and 2B4. (**a**) Total lysates of primary expanded NK cells from representative normal or XLP1 patient donors were immunoblotted for SAP and actin. (**b**,**c**) Primary rested NK cells after expansion from normal or XLP1 patient donors were mixed with 221 or K562 cells in the presence of fluorochrome-conjugated anti-CD107a mAb for degranulation assay. After incubation for 2 h, cells were stained with fluorochrome-conjugated mAb to CD56, and the level of CD56^+^CD107a^+^ NK cells was measured using flow cytometry. (**b**) Representative result is shown. (**c**) Percent increase of CD107a^+^ NK cells obtained from normal or XLP1 donors after stimulation with target cells relative to CD107a^+^ NK cells without target cells (ΔCD107a^+^ cells). Values represent the mean±s.e.m. (**d**) Primary rested NK cells after expansion from normal or XLP1 patient donors were stimulated with the indicated receptors for 5 min. Lysates were immunoblotted for the indicated phosphorylations of p65. The normalized intensities of the phosphorylated p65 relative to p65 are presented. Representative result (left) and statistical bar charts (right) are shown. Values represent mean±s.e.m. (**e**,**f**) Primary rested NK cells after expansion from normal or XLP1 patient donors were mixed with P815 target cells as indicated. After incubation for 6 h, cells were stained with fluorochrome-conjugated mAb to CD56 and analysed by flow cytometry after intracellular staining of IFN-γ. (**e**) Representative result is shown. (**f**) Percent increase of IFN-γ^+^ NK cells from individual normal or XLP1 patient donors after stimulation with the indicated receptors relative to IFN-γ^+^ NK cells without stimulation (ΔIFN-γ^+^ cells) is presented. Values represent mean±s.d. *P<0.05; **P<0.01; ***P<0.001 (two-sided Student's *t*-test). Statistical bar charts in **d** show pooled data from three different donors.

Given these promising results, NK cells from XLP1 patients were used to study the dependence of synergistic coactivation on SAP expression. Among the four XLP1 patients examined, three patients harboured macrodeletions in the *SH2D1A* gene that resulted in complete loss of SAP expression, and one patient harboured a missense mutation that reduced SAP expression, as assessed by western blot analysis (Fig. 5a and Supplementary Fig. 12). To probe the functional defects in XLP1 NK cells, we measured target cell-induced degranulation, as determined by cell surface expression of CD107a (ref. 28). Stimulation with K562 cell line induced strong degranulation of both normal and XLP1 NK cells (Fig. 5b,c). In contrast, XLP1 NK cells were severely impaired in their ability to degranulate against Epstein–Barr virus (EBV)-immortalized B-lymphoblastoid cell line 721.221 (referred to as 221), an observation compatible with the defective killing of EBV-infected B cells by XLP1 NK cells^32^.

**Figure 6:**
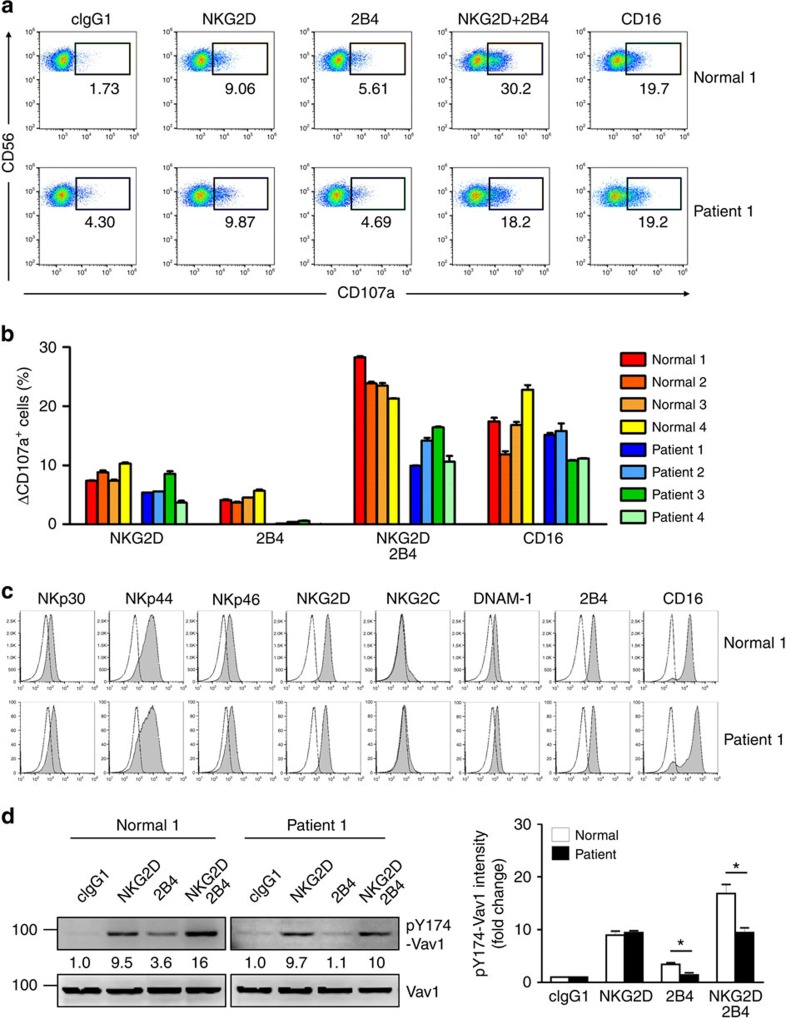
Defective cytotoxic degranulation and Vav1 activation in XLP1 NK cells following coactivation. (**a**,**b**) Primary rested NK cells after expansion from normal or XLP1 donors were mixed with P815 target cells as indicated in the presence of fluorochrome-conjugated anti-CD107a mAb. After incubation for 2 h, cells were analysed using flow cytometry as described in Fig. 5b. (**a**) Representative result is shown. (**b**) Percent increase of CD107a^+^ NK cells obtained from individual normal or XLP1 donors after stimulation with the indicated receptors relative to CD107a^+^ NK cells without stimulation (ΔCD107a^+^ cells). Values represent mean±s.d. (**c**) Representative FACS profiles showing the expression levels of the NKp30, NKp44, NKp46, NKG2D, NKG2C, DNAM-1, 2B4 and CD16 receptors (shaded histogram) on primary expanded NK cells obtained from normal or XLP1 donors. Isotype control staining is shown as the solid lines. (**d**) Primary rested NK cells after expansion from normal or XLP1 patient donors were treated as in Fig. 5d to stimulate NKG2D and/or 2B4 for 2 min. Lysates were immunoblotted with anti-pY174-Vav1 Ab and reprobed for Vav1. The normalized intensities of the phosphorylated Vav1 relative to total Vav1 are presented. Representative result (left) and statistical bar charts for pooled data from three different donors (right) are shown. Values represent mean±s.e.m. *P<0.05 (two-sided Student's *t*-test).

Next, we tested whether SAP is required for NF-κB activation and NK cell responses in XLP1 NK cells. Notably, the apparent dual phosphorylation of p65 at serine 536 and 276 following NKG2D and 2B4 coactivation seen in normal NK cells was significantly diminished in XLP1 NK cells (Fig. 5d). The synergistic phosphorylation of Erk following coactivation was also diminished in XLP1 NK cells, whereas Akt phosphorylation induced by NKG2D appeared to be unaffected. In contrast, dual phosphorylation of p65 by CD16 ligation was comparable in normal and XLP1 NK cells (Fig. 5d). Moreover, NF-κB activation in XLP1 NK cells was measured by nuclear translocation of p65 in individual NK-target cell conjugates. A significant defect in nuclear translocation of p65 following coactivation was detected in XLP1 NK cells relative to normal NK cells (Supplementary Fig. 13). Corroborating these findings, the proportion of NK cells expressing IFN-γ was severely decreased in XLP1 NK cells following NKG2D and 2B4 coactivation, but not CD16 (Fig. 5e,f). Most strikingly, the small increase in IFN-γ expression by 2B4 ligation was selectively defective in XLP1 NK cells, consistent with a critical role of SAP in 2B4-mediated signalling. A similar but less severe defect was observed when degranulation was induced through 2B4 alone and in combination with NKG2D, but not through CD16 (Fig. 6a,b). These results did not appear to be associated with defective expression of coactivation receptors in XLP1 NK cells, given comparable expression of various activating receptors, including NKG2D, 2B4 and CD16 (Fig. 6c). Instead, our results suggest that the functional deficiencies in XLP1 NK cells by NKG2D and 2B4 coactivation are most likely due to selective defects in 2B4-associated synergistic signalling to Erk, which is important for optimal NF-κB activation and effector functions.

Enhanced Vav1 signalling during NKG2D and 2B4 coactivation is required to overcome inhibition by c-Cbl and deliver synergistic signals for NK cell activation^4^. Thus, we tested whether aberrant Vav1 regulation is associated with such defects seen in XLP1 NK cells. As reported, Vav1 phosphorylation was induced in normal NK cells by engaging NKG2D or 2B4, and was additive after co-engagement (Fig. 6d). Notably, Vav1 phosphorylation was impaired in XLP1 NK cells following 2B4 stimulation, but normal after NKG2D stimulation. Furthermore, Vav1 phosphorylation after co-engaging NKG2D and 2B4 was defective in XLP1 NK cells, similar to the level of phosphorylation induced by NKG2D alone (Fig. 6d). Collectively, SAP deficiency could cause a defect in 2B4, but not NKG2D, signalling at the level of Vav1 and thereby impair the synergistic activation of Erk and NF-κB following coactivation.

### Vav1 is required for the synergistic activation of NF-κB

**Figure 7:**
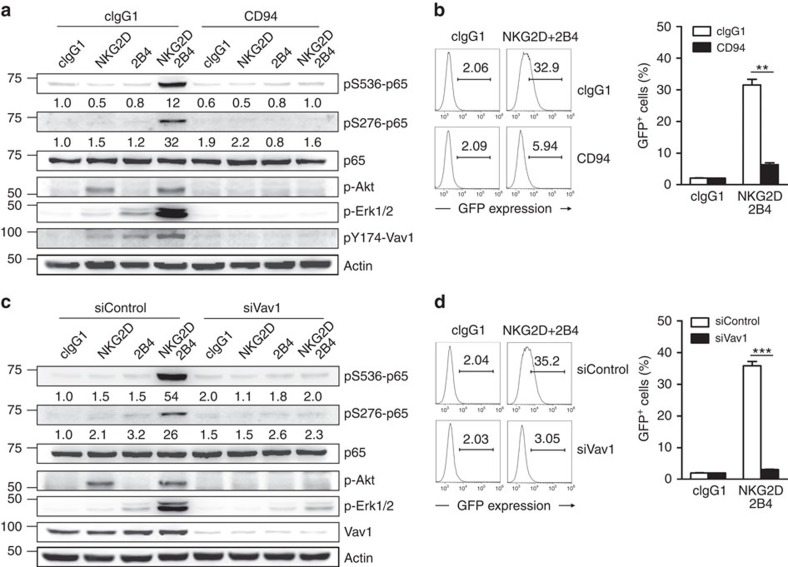
Synergistic activation of NF-κB is Vav1-dependent. (**a**) Rested NKL cells were stimulated with NKG2D and/or 2B4 in combination with or without CD94 engagement by receptor crosslinking. Lysates were immunoblotted for pS536-p65, pS276-p65, p65, p-Akt, p-Erk1/2, pY174-Vav1 or actin. The normalized intensities of the phosphorylated p65 relative to p65 are presented. (**b**) Rested NKL-κB-GFP cells were stimulated with plate-immobilized NKG2D and 2B4 in combination with or without CD94 for 6 h. GFP expression in the reporter NKL cells was analysed by flow cytometry, and representative result (left) and statistical bar charts (right) are shown. Values represent mean±s.d. (**c**) Rested NKL cells transfected with control siRNA or Vav1-specific siRNA were stimulated with NKG2D and/or 2B4 by receptor crosslinking. Lysates were immunoblotted for pS536-p65, pS276-p65, p65, p-Akt, p-Erk1/2, Vav1 or actin. The normalized intensities of the phosphorylated p65 relative to p65 are presented. (**d**) Rested NKL-κB-GFP cells transfected with control siRNA or Vav1-specific siRNA were stimulated with plate-immobilized NKG2D and 2B4 for 6 h. GFP expression in the reporter NKL cells was analysed by flow cytometry, and representative result (left) and statistical bar charts (right) are shown. Values represent mean±s.d. **P<0.01; ***P<0.001 (two-sided Student's *t*-test). Data are representative of at least three independent experiments.

To ensure that Vav1 is required for synergistic NF-κB activation, we tested whether NF-κB activation following coactivation is susceptible to Vav1 inhibition. Inhibitory signalling through CD94-NKG2A can override Vav1-dependent activation of NK cells^4^. The synergistic phosphorylation of p65 and Erk and the combined phosphorylation of Vav1 induced by NKG2D and 2B4 coactivation were all abrogated by co-crosslinking CD94-NKG2A on NKL cells (Fig. 7a). Moreover, NKG2D-dependent phosphorylation of Akt, required for pS536-p65, was also abrogated by the same inhibition. Accordingly, the synergistic increase in transcriptional activity of NF-κB was markedly impaired by co-crosslinking CD94-NKG2A (Fig. 7b). These results suggest the suppression of NF-κB activation as a mechanism underlying the inhibitory function of CD94-NKG2A. To ascertain the direct involvement of Vav1 in NF-κB activation, we performed siRNA-mediated knockdown of Vav1. Similarly, Vav1 knockdown abrogated the phosphorylation of p65, along with that of Akt and Erk, and in turn transcriptional activity of NF-κB following coactivation (Fig. 7c,d). Collectively, our results suggest that NF-κB activation in NK cells by NKG2D and 2B4 coactivation is under the control of Vav1 and dominantly inhibited by CD94-NKG2A.

## Discussion

**Figure 8:**
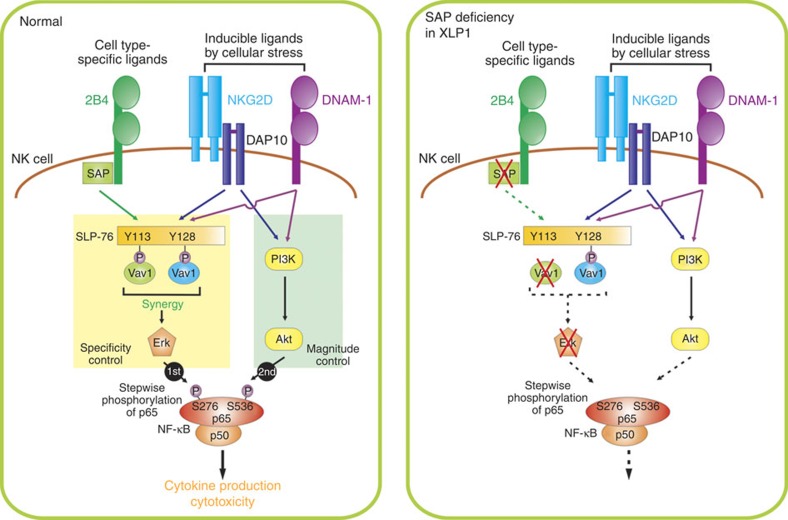
Proposed mechanism of NF-κB activation via coactivation receptors on NK cells. NF-κB activation in NK cells required the coordinated engagement of coactivation receptors, such as 2B4 and NKG2D or 2B4 and DNAM-1. This combination was required to provide complementary and independent signals leading to Vav1-dependent synergistic signalling involving PLC-γ2 and Erk. Further, signals from synergizing receptors converged on NF-κB p65 subunit through selective phosphorylation of p65 serine residues, particularly at serine 276 via Vav1-Erk and at serine 536 via PI3K-Akt pathway, which was crucial to optimal activation of NF-κB. The requisite PI3K-Akt signal was primarily mediated by the engagement of NKG2D or DNAM-1, which recognizes ligands induced by cellular stress. Vav1 controlled downstream p65 phosphorylation and NF-κB activation, suggesting that distinct signalling checkpoints at the level of Vav1 and p65 regulate NF-κB activation. In support, Vav1-dependent synergistic signalling was required for the phosphorylation of p65 at serine 536 by Akt pathway, which was evident in SAP-deficient XLP1 NK cells following coactivation, which exhibited impaired p65 phosphorylation, nuclear translocation, NF-κB activation and downstream NF-κB-dependent functions.

Here we offer a new perspective on the regulation of NF-κB activation in NK cells during target cell recognition. NF-κB activation via non-ITAM-associated receptors (for example, NKG2D, 2B4, DNAM-1) relies on coordinated engagement of coactivation receptors, which together provide complementary and independent signals leading to optimal Vav1 and p65 phosphorylation (Fig. 8). The importance of such regulation between Vav1 and p65 in NF-κB activation was supported by signalling and functional defects centred on Vav1 in XLP1 NK cells following coactivation.

A prevailing view of the mechanism underlying synergistic coactivation involves signal integration by receptor-proximal adaptor proteins that promote downstream signalling events for cytokine production and target cell killing^1^. Supporting this, it was shown that signals from synergizing receptors converge on the adaptor protein SH2 domain-containing leukocyte phosphoprotein of 76 kDa (SLP-76) through site-selective phosphorylation of two tyrosines in SLP-76 (ref. 34). These two phosphotyrosines enable simultaneous binding of Vav1-Nck protein complex to both tyrosines on SLP-76, which leads to Vav1-dependent synergistic signals. Vav1 is an essential component for synergistic coactivation among combinations of NKG2D, 2B4 and DNAM-1 (ref. 4). Thus, the dependence of synergistic coactivation on the regulated interaction between SLP-76 and Vav1 may represent a checkpoint in NK cell activation that ensures proper specificity of NK cell responses. Supporting this notion, the defective phosphorylation of Vav1 in XLP1 NK cells, and the inhibition of Vav1 by inhibitory receptor or Vav1 knockdown resulted in abrogation of NF-κB activation during coactivation.

In addition, our study revealed that Vav1-dependent synergistic signalling was crucial but insufficient for full NF-κB activation. The outcome of NF-κB activation also relied on signal integration by NF-κB p65 subunit via specific phosphorylation at regulatory serine residues. Post-translational modification of NF-κB subunit has long been appreciated as an important regulatory mechanism, dictating its transcriptional activity and target gene specificity^25^. Among others, phosphorylation of the p65 subunit plays a key role in determining the specificity, strength and duration of NF-κB-dependent gene programmes^24^,^27^. Thus, such modification likely serves to fine-tune NF-κB transcriptional activity, rather than functions as a simple on-off switch^27^. Although the possibility of signal integration by other proteins cannot be excluded, our results reveal that synergistic NF-κB activation is kept in check at the level of p65 by the requirement for a PI3K-Akt signal, in addition to the Vav1-Erk signal. In a T-cell study, Akt was shown to fine-tune NF-κB signalling and transcription during CD3 and CD28 stimulation, in part through its effects on p65 (ref. 35). Of interest, we observed that the requisite PI3K-Akt signal for NF-κB activation was principally mediated by stimulation through NKG2D or DNAM-1, but not 2B4. The natural ligands for NKG2D (MICA/B and the family of ULBPs) and DNAM-1 (CD155 and CD112) are frequently upregulated on cells under stress conditions associated with malignant transformation or viral infection^36^,^37^. Thus, it is likely that NK cell stimulation by NKG2D or DNAM-1 in combination with 2B4 could trigger tailored NF-κB responses according to the expression levels of cognate ligands on stressed cells.

The regulation of NF-κB activation at multiple levels may serve as a safeguard to prevent inadvertent gene transcription. The identification of distinct signalling checkpoints ‘upstream' at SLP-76-Vav1 and ‘downstream' at p65, as shown here, is consistent with the tight control of NF-κB activation. Similar patterns of SLP-76, Vav1 and p65 phosphorylation were induced by synergy among NKG2D, 2B4 and DNAM-1, suggesting a common logic for signal coordination among coactivation receptors. Moreover, the dependence of synergistic p65 phosphorylation and NF-κB activation on ‘upstream' Vav1, and the defects of such regulation in XLP1 NK cells, suggests that stepwise signalling checkpoints at the level of Vav1 and p65 control NF-κB activation. Supporting this, p65 phosphorylation at serine 536 by PI3K-Akt pathway was apparent upon stimulation with NKG2D and 2B4, but not NKG2D alone, although Akt phosphorylation by NKG2D crosslinking was not enhanced by co-crosslinking with 2B4. Likewise, in XLP1 NK cells, Vav1-dependent synergistic signalling was prerequisite to mediate pS536-p65 by Akt pathway.

Our present analysis of XLP1 NK cells provides an insight into the mechanism by which SAP deficiency affects NF-κB activation and NK cell functions during coactivation. Mutations in the *SH2D1A* gene, which result in the lack or dysfunction of SAP, form the genetic basis of XLP1 (refs 29, 30, 31). XLP1 patients are particularly susceptible to EBV infection. Among the defects in XLP1 lymphocytes, the inability of XLP1 NK and CD8^+^ T cells to eliminate EBV-infected B cells largely accounts for the persistence of infected B cells, fulminant mononucleosis and B-cell lymphoma^38^,^39^. SAP is an adaptor protein required for transmitting activation signals elicited through SLAM family receptors, including 2B4 (ref. 40). Accordingly, SAP-deficient XLP1 NK cells fail to be activated through 2B4 and show defects in 2B4-mediated killing of EBV-infected B cells and production of IFN-γ^32^,^33^. Here we found that SAP deficiency impedes synergistic NF-κB activation by NKG2D and 2B4 coactivation at the level of Vav1 and Vav1-dependent downstream signals, such as Erk. This Erk pathway activation was required for cytotoxic degranulation and crucial to phosphorylate p65 at key serine residue (S276-p65) for NF-κB activation. These results are consistent with the dependence of 2B4-mediated activation on SAP through Fyn-induced phosphorylation of Vav1 (ref. 8). However, possible contribution of SAP deficiency to other transcription factors including IRFs cannot be excluded. In SAP deficiency, 2B4 was shown to recruit protein tyrosine phosphatases (for example, SHIP-1) and impair the activity of co-engaged activating receptors by delivering inhibitory signals^8^,^30^,^32^. A recent study showed that this inhibitory function of 2B4 in XLP1 NK cells is confined to ITAM-dependent signalling pathways and does not affect the activity of non-ITAM-associated NKG2D and DNAM-1 (ref. 41). In support, upon NKG2D and 2B4 co-engagement, NKG2D-dependent phosphorylation of Vav1 and Akt were preserved, whereas synergistic signals through combined Vav1 phosphorylation were abrogated in XLP1 NK cells (Figs 5 and 6). In SAP-null murine NK cells, 2B4 could repress NKG2D^42^, which, unlike human NKG2D, recruits both DAP10 and ITAM-associated DAP12 (ref. 23). Thus, we speculate that 2B4-mediated inhibition is selective to the activating receptors co-engaged, although the exact mechanism underlying this selectivity in inhibition remains to be determined.

It has been shown that NK cells become ‘primed' upon exposure to cytokines such as IL-2 or IL-15 and, in turn, have enhanced reactivity against target cells, a situation that likely occurs during the course of infection and transformation. Such cytokine-stimulated NK cells respond to the engagement of single activating receptor (for example, NKG2D, 2B4) for effector functions^5^,^6^,^28^,^43^, probably due to a lower threshold for activation than resting NK cells. Supporting this, the proportion of cells expressing IFN-γ was significantly increased in IL-2 stimulated NK cells following stimulation with NKG2D or 2B4 alone, correlating with enhanced NF-κB activation (Supplementary Fig. 14a,b). Further, IFN-γ expression and NF-κB activation by NKG2D and 2B4 co-engagement were also enhanced after IL-2 stimulation, suggesting that synergistic coactivation exists, even in the context of a high IL-2 environment. We found a gradual decrease in c-Cbl but not Vav1 and a marginal phosphorylation of Vav1 after IL-2 stimulation (Supplementary Fig. 14c,d), suggesting downregulation of c-Cbl as a potential mechanism that relieves the requirement for coactivation. However, the involvement of other regulatory mechanism(s) cannot be excluded.

In conclusion, we provide evidence that, unlike ITAM-dependent pathways, such as those triggered by antigen-specific receptors of adaptive immune cells and Fcγ receptor CD16 in NK cells, a single coactivation receptor such as 2B4 or NKG2D is incompetent to induce NF-κB activation. Instead, it requires complementation of coactivation receptors with distinct signalling properties to achieve proper specificity and optimal magnitude of NF-κB activation. Because PI3K-Akt and Erk pathways are often induced by diverse NF-κB-activating stimuli^26^,^27^ and involved in site-selective phosphorylation of p65, the model for coordinated NF-κB activation through combined p65 phosphorylation described here may apply to other NF-κB-activating stimuli in various cell types.

## Methods

### Cells and reagents

Human blood samples from normal healthy donors and XLP1 donors were drawn for research purposes under a protocol approved by the institutional review board with informed consent. Patients with a confirmed *SH2D1A* gene mutation were included. Peripheral blood mononuclear cells (PBMCs) were isolated from the blood samples by density gradient centrifugation (LSM lymphocyte separation medium; MP Biomedicals) and cryopreserved until processed. Human NK cells were purified from PBMCs by negative selection using the NK cell isolation kit (StemCell Technologies). These cells were 97–99% CD3-CD56^+^, as assessed by flow cytometry. The human NK cell line NKL (gift from M. Robertson) was cultured in RPMI-1640 supplemented with 10% FBS, 1 mM sodium pyruvate and 200 U ml^−1^ recombinant IL-2 (rIL-2). NKL cells were rested in RPMI-1640 supplemented with 5% FBS and 0.5 mM sodium pyruvate without rIL-2 for 24 h. P815 cells (American Type Culture Collection) were cultured in IMDM (Cellgro) supplemented with 10% FBS and 2 mM  L-glutamine. P815 cells are FcR^+^ and, upon incubation with antibodies (Abs) to NK cell-activating receptors (for example, NKG2D and 2B4), bind the Abs via the Fc-region. In doing so, they can activate NK cells through specific activating receptors in direct cell–cell contact. 721.221 cells (221; gift of J. Gumperz and P. Parham) and K562 cells (American Type Culture Collection) were maintained in IMDM (Cellgro) supplemented with 10% FBS. K562 cells are of the erythroleukemia type and known to express ligands for NKG2D, DNAM-1 and NKp30 receptors^44^,^45^. 221 cells are an EBV-transformed B cell line, and their lysis by NK cells is associated with 2B4, NKp44 and NKp46 receptors^32^,^46^. K562 and 221 cells were used to validate the functional defects of XLP1 NK cells, given an impaired susceptibility of 221 cells to XLP1 NK cells likely due to the involvement of 2B4 in mediating lysis of the cells. K562-mb15-41BBL cell line (gift of D. Campana) for NK cell expansion were cultured in RPMI-1640 supplemented with 10% FBS. Expression of human ligands for NK cell receptor in P815 cells has been described^47^. 293T cells were cultured in DMEM (Cellgro) supplemented with 10% FBS and 2 mM GlutaMAX (Gibco). The cells were free of mycoplasma contamination. All chemicals were from Calbiochem unless indicated otherwise.

### Patient samples

Four patients with a confirmed mutation in the *SH2D1A* gene were included in this study. XLP1 patient samples (patients 1 and 2) were provided by Dr Booth^48^. Patient samples 3 and 4 were obtained from Dr Nichols^49^. Both patients 1 and 2 had macrodeletions in the *SH2D1A* gene. Patient 3 exhibited a T53I missense mutation in the *SH2D1A* protein and patient 4 had a germline deletion in the *SH2D1A* gene. The expression of SAP protein was absent in the NK cells obtained from three patients (patients 1, 2 and 4) and very low in patient 3′s NK cells.

### Abs

Abs for NK cell receptors and signalling molecules were obtained from the following sources: NKG2D (149810; R&D Systems); CD244/2B4 (C1.7; Beckman Coulter); isotype control mouse IgG1 (MOPC-21; Sigma); CD226/DNAM-1 (DX11), CD16 (3G8), CD94 (HP-3D9), pS32/36 IκBα (39A1413) and actin (C4) (BD Biosciences); pY174 Vav1 (ab47282) and pS276 p65 (ab30623; Abcam); Vav1 (H211), p65 (F-6), p50 (H-119), SAP (1D12) and IκBα (C-21; Santa Cruz); α-tubulin (GT114; GeneTex); Vav1 (R775), pS536 p65 (93H1), pS176/180 IKKα/β (16A6), pS473 Akt (9271), pY202/204 Erk1/2 (9101) and TBP (44059; Cell Signaling); c-Cbl (7G10) and T7 (AB3790; Millipore). Goat F(ab′)2 anti-mouse IgG was obtained from Jackson ImmunoResearch. The fluorochrome-conjugated Abs were used in the flow cytometric analyses: α-pS536 p65-Alexa Fluor 488 (93H1) and isotype control rabbit IgG-Alexa Fluor 488 (DA1E; Cell Signaling); α-CD3-PerCP (SK7), α-CD56-PE (NCAM16.2), α-CD107a-FITC (H4A3), α-IFN-γ-FITC (25723.11), α-CD16-PE (3G8), α-CD336/NKp44-PE (p44-8.1), α-NKp46-PE (9E2), α-CD226/DNAM-1-PE (DX11) and α-CD48-PE (TÜ145; BD Bioscience); α-TNF-α-FITC (MAb11, eBioscience); α-CD244/2B4-PE (C1.7) and α-NKp30-PE (Z25; Beckman Coulter); α-NKG2D-PE (149810), α-NKG2C-PE (134591) and α-ULBP1-PE (170818; R&D Systems). CFSE, α-mouse IgG-Biotin and Alexa Fluor 647-Streptavidin were obtained from Invitrogen for use in confocal microscopy. Mouse Fc block was done with anti-Fcγ RII/III (2.4G2; BD Bioscience).

### Cellular assays

IFN-γ (Pierce) and MIP-1α (R&D Systems) production after stimulation of cells with beads coated with monoclonal antibodies (mAbs) to NK receptors were determined by ELISA. Briefly, beads for NK cell stimulation were prepared by incubating goat anti-mouse-coated beads (4 × 10^7^; Dynabeads M-450) with the indicated mAbs (3 μg) in PBS containing 2% FBS for 1 h at 4 °C. After washing three times in PBS, 2% FBS, the beads (4 × 10^7^) were incubated with rested NKL cells (4 × 10^7^) in 500 μL IMDM, 10% FBS for the indicated times at 37 °C. The cultures were rotated end-over-end during the stimulation, after which the supernatants were assayed.

Intracellular Ca^2+^ mobilization was measured by flow cytometry in cells labelled with Fluo-4 AM (Invitrogen) as described^34^. Briefly, NK cells were labelled for 30 min at 30 °C with dye-loading buffer (Hanks' balanced salt solution (HBSS) with 1% FBS, 2 μM Fluo-4 AM and 4 mM probenecid). Cells were then washed twice, resuspended in HBSS, 1% FBS and incubated with the indicated mAbs (10 μg ml^−1^) for 30 min on ice. Cells were washed twice and resuspended in HBSS containing 1% FBS, and transferred to flow cytometry analysis tubes. Cells were warmed for 5 min at 37° in a water bath, and placed on the flow cytometer. After 30 s of data acquisition, tubes were removed, and 4 μg of cross-linking goat anti-mouse F(ab′)_2_ was added. Cells were mixed by vortexing, placed back on the flow cytometer (BD Bioscience) and events were acquired for a further 5 min. Data were analysed with FlowJo software (Tree Star).

Degranulation of NK cells was assessed by CD107a expression on the cell surface and granzyme B release (BioLegend) as described^28^. Briefly, primary rested NK cells were mixed with an equal number of K562 or 221 cells in the presence of anti-CD107a-FITC mAbs, spun down for 3 min at 30*g*, incubated for 2 h at 37 °C, and spun down again. The cell pellets were resuspended in FACS buffer (PBS with 2% FBS) and stained with anti-CD56-PE for 30 min in the dark at 4 °C. Lymphocytes were gated on forward scatter/side scatter, and the CD107a expression on NK cells was analysed by flow cytometry and FlowJo software. For granzyme B release assay, rested NKL cells after transfection with control siRNA or p65-specific siRNA were stimulated with plate-immobilized mAbs to NKG2D and/or 2B4 for 2 h. Thereafter, granzyme B release into the supernatants were determined by ELISA.

Cytokine production by primary NK cells was determined by the intracellular expression of IFN-γ and TNF-α as described^14^. PBMCs or primary rested NK cells were stimulated with an equal number of the indicated target cells for 1 h at 37 °C. Thereafter, brefeldin A (GolgiPlug; BD Bioscience) was added, followed by an additional 5 h of incubation for a total of 6 h. The cells were then stained for surface markers with anti-CD56-PE and/or anti-CD3-PerCP mAbs for 30 min in the dark at 4 °C. After washing the cells twice with FACS buffer, they were incubated in BD Cytofix/Cytoperm solution (BD Bioscience) for 20 min in the dark at 4 °C. Before and after intracellular staining with anti-IFN-γ-FITC or anti-TNF-α-FITC mAb for 30 min in the dark at 4 °C, the cells were washed twice with BD Perm/Wash buffer (BD Bioscience). The cells were then analysed by flow cytometry gated on NK cells.

### Receptor crosslinking and cell mixing experiments

For Ab-mediated crosslinking of NK receptors, NK cells were preincubated with isotype control mAb or mAbs specific for NK receptors (all at 10 μg ml^−1^) for 30 min on ice. After washing unbound Abs with medium, NK cells were stimulated by crosslinking with 30 μg ml^−1^ goat anti-mouse F(ab′)_2_ secondary Ab at 37 °C for the indicated times. For stimulation of NK receptors mediated by plate-immobilized Abs, 96-well Costar EIA/RIA Stripwell plates were coated overnight at 4 °C with isotype control mAb or mAbs specific for NK receptors (all at 10 μg ml^−1^). Then, NK cells were added into NK receptor mAb-coated plates for the indicated times. For cell mixing experiments, NK and P815 target cells separately chilled on ice were mixed at an effector to target ratio of 1:1. Cells were incubated for 10 min on ice and then incubated at 37 °C for the indicated times. Cells were moved to ice and then lysed for further analysis.

### Assessment of NF-κB activation by electrophoretic mobility shift assay (EMSA)

Nuclear and cytoplasmic fractions were isolated from stimulated and unstimulated NKL cells using Nuclear Extract kit (Active Motif) according to the manufacturer's protocol. The amount of p65 in nuclear fraction and its DNA-binding activity were measured by an immunoblotting and TransAM NF-κB p65 ELISA kit (Active Motif) following the manufacturer's instruction, respectively.

### NF-κB reporter assay

NKL reporter cells (NKL-κB-GFP) that express GFP under the control of NF-κB transcription response elements were generated by transducing NKL cells with lentiviral κB-GFP construct (System Biosciences). Lentiviral particles were produced by simultaneous transfection of 293TN packaging cell line (System Biosciences) with pGreenFire-NF-κB-EF1-Puro vector and pPACKH1 packaging plasmid mix (System Biosciences). NKL cells were transduced with lentivirus supernatant that encodes κB-GFP in the presence of 10 μg ml^−1^ polybrene and 200 U ml^−1^ rIL-2 and then selected with 1 μg ml^−1^ puromycin (2 days after transduction). Thereafter, NKL transductants showing GFP upregulation upon TNF-α treatment were further selected by FACS-sorting and grown as pure cultures. To assess NF-κB activation, NKL-κB-GFP cells were stimulated with TNF-α or plate-immobilized mAbs specific for NK receptors, and GFP expression in the reporter NKL cells was analysed by flow cytometry.

### RNA interference

NKL cells were transfected with 300 pmol of siRNA with the Amaxa Nucleofector II system. A total of 1.2 × 10^6^ cells were resuspended in 100 μl of Amaxa kit solution V (Lonza), mixed with siRNA and immediately transfected with programme O-017. For a total of 48 h incubation at 37 °C, cells were rested for the last 24 h and then assayed as indicated. For knockdown in primary NK cells, 1.5 × 10^6^ expanded cells were resuspended in 100 μl of Amaxa kit solution for human macrophage, mixed with 300 pmol of siRNA and transfected with programme X-001. Thereafter, cells were incubated for 36 h in the presence of IL-2 (200 U ml^−1^), rested for 12 h and then assayed as indicated. The siRNAs specific for NF-κB p65 were part of TriFECTa Dicer-substrate kit obtained from Integrated DNA Technologies (IDT) and the following siRNA sequences were used: p65, 5′- GGAGUACCCUGAGGCUAUAACUCGC -3′ (sense) and 5′- GCGAGUUAUAGCCUCAGGGUACUCCAU -3′ (antisense) or 5′- GGACAUAUGAGACCUUCAAGAGCAT -3′ (sense) and 5′- AUGCUCUUGAAGGUCUCAUAUGUCCUU -3′ (antisense). The siRNAs used to knockdown of Vav1 and c-Cbl were described previously^4^ and the sequences are as follows: Vav1, 5′- CGUCGAGGUCAAGCACAUUdTdT -3′ (sense) and 5′- AAUGUGCUUGACCUCGACGdTdT -3′ (antisense); c-Cbl: 5′- CCUCUCUUCCAAGCACUGAdTdT -3′ (sense) and 5′- UCAGUGCUUGGAAGAGAGGdTdT -3′ (antisense). ON-TARGETplus SMARTpool siRNAs specific for Vav1 (L-003935), c-Cbl (L-003003) were also obtained from Dharmacon. The siRNA specific for SAP were obtained from IDT and the sequences are as follows: SAP, 5′- GCUGUAUCACGGUUACAUUUAUACA -3′ (sense) and 5′- UGUAUAAAUGUAACCGUGAUACAGCAC -3′ (antisense). siRNA specific for SAP (SI00036561) were also obtained from Qiagen. ON-TARGETplus SMARTpool siRNAs specific for Akt1 (L-003000) or Erk2 (L-003555) were obtained from Dharmacon. A second set of siRNAs for Akt1 and Erk2 was used, with the following sequences: Akt1, 5′- GGACAGAGGAGCAAGGUUUdTdT -3′ (sense) and 5′- AAACCUUGCUCCUCUGUCCdTdT -3′ (antisense); Erk2: 5′- GGGUUCCUGACAGAAUAUGdTdT -3′ (sense) and 5′- CAUAUUCUGUCAGGAACCCdTdT -3′ (antisense). Similar results were obtained with either one of the siRNAs used. The results shown in this paper are those obtained with the former set of siRNA oligonucelotides. The negative siRNA controls were obtained from IDT and Dharmacon.

### *Ex vivo* expansion of NK cells

Owing to the limited supply of patient NK cells, NK cells from XLP1 patients were expanded to study molecular signals for NF-κB activation and effector functions. NK cells from normal donors were also expanded and, after a period of rest, reproduced the synergistic increase in effector functions and signalling following stimulation with NKG2D and 2B4. Primary NK cells obtained from normal or XLP1 donors were expanded by stimulation with the K562-mb15-41BBL cell line, as previously described^50^,^51^ with slight modifications. 1.5 × 10^6^ PBMCs were cultured with 1 × 10^6^ 100 Gy gamma ray-irradiated K562-mb15-41BBL feeder cells in stem cell growth medium (CellGenix) supplemented with 10% FBS and 10 U ml^−1^ rIL-2. The medium was changed every 2 days and replaced with fresh medium containing 10 U ml^−1^ rIL-2. After 1 week of coculture, residual T cells were depleted using the CD3^+^ selection kit (StemCell Technologies). The remaining cells were further expanded for an additional 2 weeks in stem cell growth medium supplemented with 10% FBS, 100 U ml^−1^ rIL-2 and 5 ng ml^−1^ rIL-15. The resulting cell population was 97–99% CD3-CD56^+^, as assessed using flow cytometry.

### DNA constructs and transfections

Given a difficulty in detecting p65 phosphorylation at serine 276 (ref. 52), the specificity of the antibody against pS276 p65 (ab30623) was confirmed (Supplementary Fig. 15). The plasmids encoding T7-RelA (#21984) or T7-RelA S276A (#24153) were obtained from Addgene and verified by sequencing. 0.5 × 10^6^ 293T cells were cultured for 18 h and then transfected with 2 μg of plasmid DNA using X-tremeGENE 9 (Roche). Medium was changed after 24 h post-transfection and transfected cells were assayed after another 24 h.

### Immunoblotting

The stimulated NK cells were washed with ice-cold PBS and lysed in lysis buffer (50 mM Tris-HCl (pH 7.5), 150 mM NaCl, 1% Triton X-100, 5 mM EDTA, 1 mM NaVO_3_, 50 mM NaF, 1 mM phenylmethylsulfonyl fluoride (PMSF) and protease inhibitor cocktail (Thermo)) for 30 min on ice. Cell debris, including the nuclei, was removed by centrifugation, and the supernatants were recovered. The protein concentration of the cell lysates was determined using the BCA protein assay kit (Pierce). Lysates were resuspended in 1 × NuPAGE LDS sample buffer (Invitrogen) containing 50 mM dithiothreitol, and further incubated for 10 min at 70 °C. Equal amounts of protein for each sample were resolved on 8% Tris-HCl gel or 4–20% Tris-HEPES gel (Thermo) and subsequently transferred onto PVDF membranes (Millipore) in transfer buffer (25 mM Tris, 192 mM glycine, 20% (v/v) methanol). The membranes were blocked with 5% BSA or skim milk in TBS-T (Tris-buffered saline containing 0.1% Tween-20) for 1 h and subsequently incubated with primary Abs and then with the HRP-conjugated secondary Abs. Blots were developed using SuperSignal West Pico and detected using LAS-4000 (Fujifilm). Images have been cropped for presentation. Full-size images are presented in Supplementary Fig. 16.

### Cell conjugation and immunofluorescence

To examine NF-κB p65 translocation into the nucleus in the individual NKL cells that were conjugated with target cells, NKL cells were first stained with 10 μM CFSE (Invitrogen) for 15 min at 37 °C, washed and resuspended in IMDM supplemented with 5% FBS. P815 cells were incubated with 10 μg ml^−1^ isotype control mAb or mAbs specific to NK receptors for 20 min at room temperature (RT), spun down and resuspended in IMDM containing 5% FBS. Thereafter, CFSE-loaded NKL cells were conjugated to the P815 target cells at a 2:1 *E*/*T* ratio for 30 min at 37 °C and then allowed to adhere to poly-L-lysine-coated slide glass (Sigma) for 15 min at 37 °C. Slides were fixed in 4% paraformaldehyde (Electron Microscopy Sciences) for 30 min at RT and in cold methanol for 10 min at −20 °C. The cells were then permeabilized with 0.1% Triton X-100 (Sigma) and 0.1% sodium citrate (Sigma) in PBS for 3 min at 4 °C and blocked with PBS containing 1% BSA and 1% goat serum for 30 min. Slides were incubated with primary Ab to p65 (1:100, F-6; Santa Cruz) for 90 min, α-mouse IgG-Biotin (1:100; Invitrogen) for 40 min, Alexa Fluor 647-Streptavidin (1:50; Invitrogen) for 30 min in PBS containing 1% BSA and 0.5% Triton X-100, and then 4,6-diamidino-2-phenylindole (300 nM; Molecular Probes) for 2 min. All slides were incubated at RT and washed three times with PBS. The slides were mounted with ProLong Gold antifade reagent (Molecular Probes) and 0.13–0.16 mm coverslips (Marienfeld). The cells were imaged using the Zeiss LSM710 laser-scanning Confocal Microscope (Carl Zeiss) and analysed using the MetaMorph software (Molecular Devices). NKL-target cell conjugates were verified by the presence of a CFSE-stained cell that had conjugated to an unstained cell.

### Real-time PCR

To assess the expression of genes related to NK cell effector functions, total RNA was isolated using the RNeasy kit (QIAGEN). cDNA was synthesized from 1 μg RNA using the ReverTra Ace qPCR RT kit (Toyobo) according to the manufacturer's instructions. Real-time PCR amplification and analysis were conducted using the SYBR Green Realtime PCR Master Mix (Toyobo) and LightCycler 480 Real-Time PCR System (Roche Diagnostics). The PCR conditions were as follows: preheating for 10 min at 95 °C, 40 cycles of 95 °C (30 s), 60 °C (30 s) and 72 °C (30 s). Melting curve analysis was done using the default settings of the device. RNA levels were normalized to β-actin expression using the ΔΔCt method. Primer sequences for real-time PCR are described in the Supplementary Table 1.

### Flow cytometric analysis of p65 phosphorylation

Purified human primary NK or NKL cells were incubated with isotype control mAb or mAbs specific to NK receptors on ice for 30 min. After being washed with medium, NK cells were stimulated by receptor cross-linking with goat anti-mouse F(ab′)2 secondary Ab at 37 °C for 5 min. The cells were washed with DPBS and fixed with 4% paraformaldehyde at 37 °C for 10 min. The fixed cells were then permeabilized with 90% methanol on ice for 30 min and blocked with 0.5% BSA at RT for 10 min. Cells were analysed for p65 phosphorylation using flow cytometry after staining with Alexa Fluor 488-conjugated anti-pS536 p65 Ab (93H1) or isotype control rabbit IgG (DA1E; Cell Signaling).

### Statistical analyses

Each graph was generated from at least three independent experiments. Results are presented as the mean±s.d. or s.e.m. Statistical analyses were conducted by two-tailed Student's *t*-test using the GraphPad Prism software.

### Data availability

The data that support the findings of this study using patient samples are available on request from the corresponding author H.S.K. The data are not publicly available due to them containing information that could compromise research participant privacy/consent.

## Additional information

**How to cite this article:** Kwon, H.-J. *et al*. Stepwise phosphorylation of p65 promotes NF-κB activation and NK cell responses during target cell recognition. *Nat. Commun.* 7:11686 doi: 10.1038/ncomms11686 (2016).

## Supplementary Material

Supplementary Information Supplementary Figures 1 - 16 and Supplementary Table 1
