# Supplementary material for: Stepwise phosphorylation of p65 promotes NF-κB activation and NK cell responses during target cell recognition
Source: Nat Commun. 2016 May 25;7:11686. doi: 10.1038/ncomms11686 (PMC4894962; doi:10.1038/ncomms11686)
Supplement: Supplementary Information — Supplementary Figures 1 - 16 and Supplementary Table 1 [file ncomms11686-s1.pdf]

Supplementary fig. 1

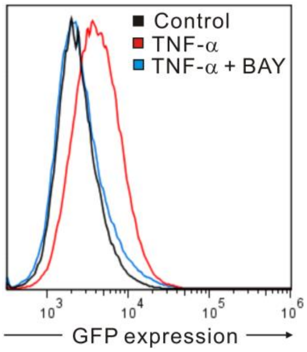

Supplementary figure 1. GFP expression in NKL-κB-GFP cells is NF-κB-dependent. Rested NKL cells transduced with the κB-GFP reporter construct were treated with 10 ng/mL TNF-α for 6 h after pretreatment with the vehicle or NF-κB inhibitor BAY11-7082 (1 μM) for 1 h. GFP expression in NKL-κB-GFP cells was analyzed using flow cytometry.

Supplementary fig. 2

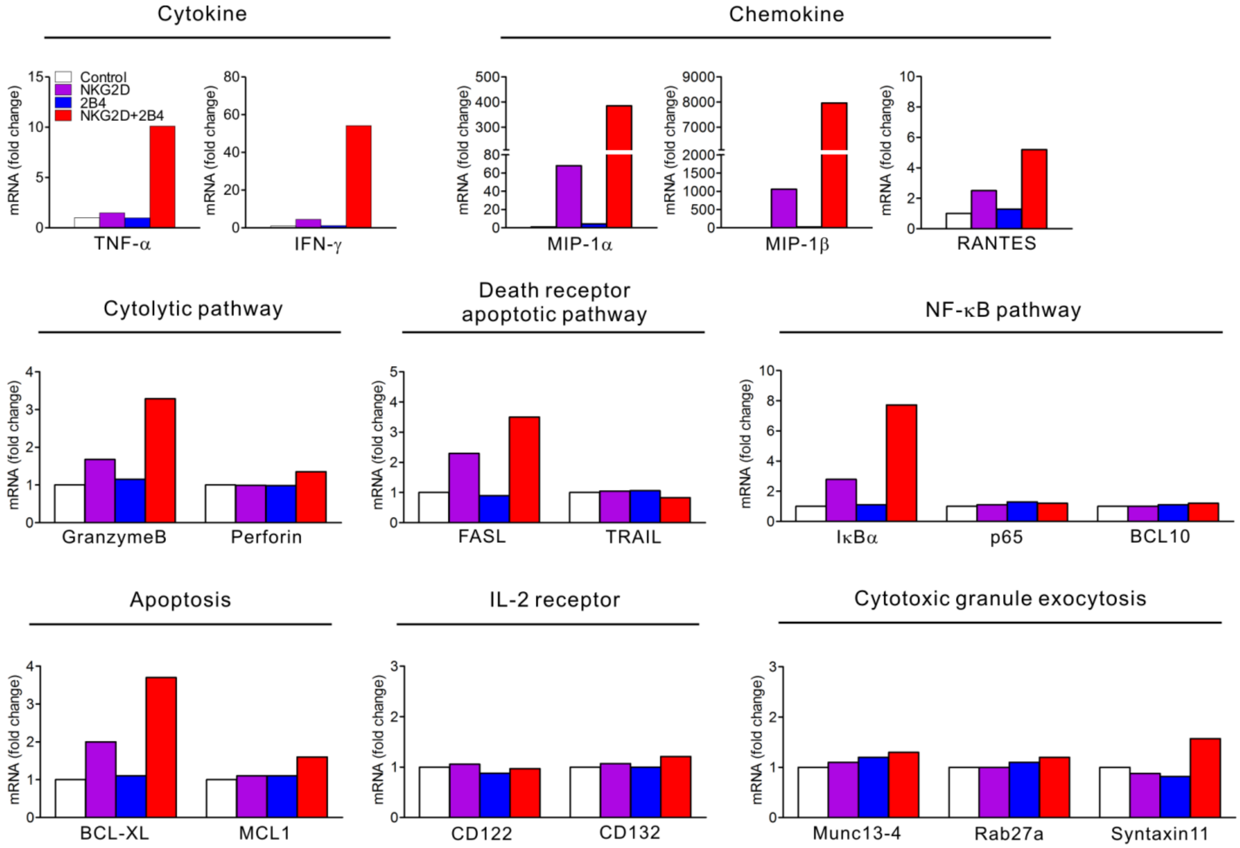

Supplementary figure 2. Profile of gene expression related to NK cell function during coactivation.

Rested NKL cells were stimulated with NKG2D and/or 2B4 for 3 h. Thereafter, total RNA was prepared from cells, reverse transcribed, and analyzed by real-time PCR with primers specific for TNF-α, IFN-γ, MIP-1α, MIP-1β, RANTES, Granzyme B, Perforin, FASL, TRAIL, IκBα, p65, BCL10, BCL-XL, MCL1, CD122, CD132, Munc13-4, Rab27a, or Syntaxin11. The relative mRNA levels were determined by real-time PCR and normalized to β-actin mRNA. Data are representative of three independent experiments.

Supplementary fig. 3

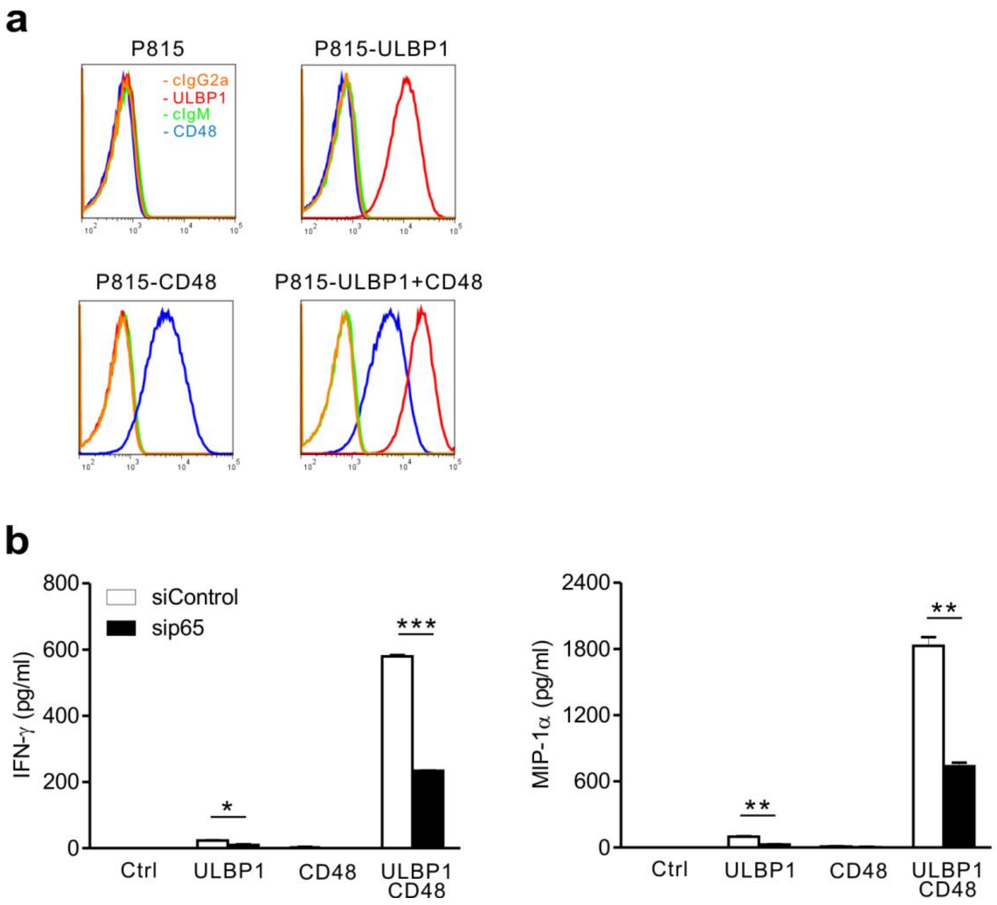

Supplementary figure 3. IFN-γ and MIP-1α production by NKG2D and 2B4 ligands is NF-κB-dependent.

(**a**) Expression of NKG2D and 2B4 ligands on transfected P815 cells. P815, P815-ULBP1, P815-CD48, and P815-ULBP1+CD48 cells were stained with isotype control mAbs, or mAbs to ULBP1 and CD48.

(**b**) Cytokine release assays with rested NKL cells transfected with control siRNA or p65-specific siRNA and stimulated with P815 cells expressing ULBP1 and/or CD48. After incubation for 6 h, IFN-γ (left) and MIP-1α (right) in the supernatants were measured by ELISA. Values represent mean ± s.d. *P<0.05; **P<0.01; ***P<0.001 (two-sided Student's t-test). Data are representative of three independent experiments.

Supplementary fig. 4

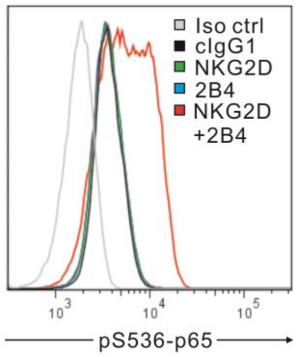

Supplementary figure 4. The proportion of cells showing p65 phosphorylation is synergistically increased by NKG2D and 2B4 coactivation.

Phosphorylation of the NF-κB p65 subunit in rested NKL cells that were stimulated with NKG2D and/or 2B4 by receptor crosslinking for 5 min. The cells were fixed, permeabilized, stained with Alexa Fluor 488-conjugated isotype control mAb or mAb specific to phospho-p65 at serine 536, and analyzed using flow cytometry.

Supplementary fig. 5

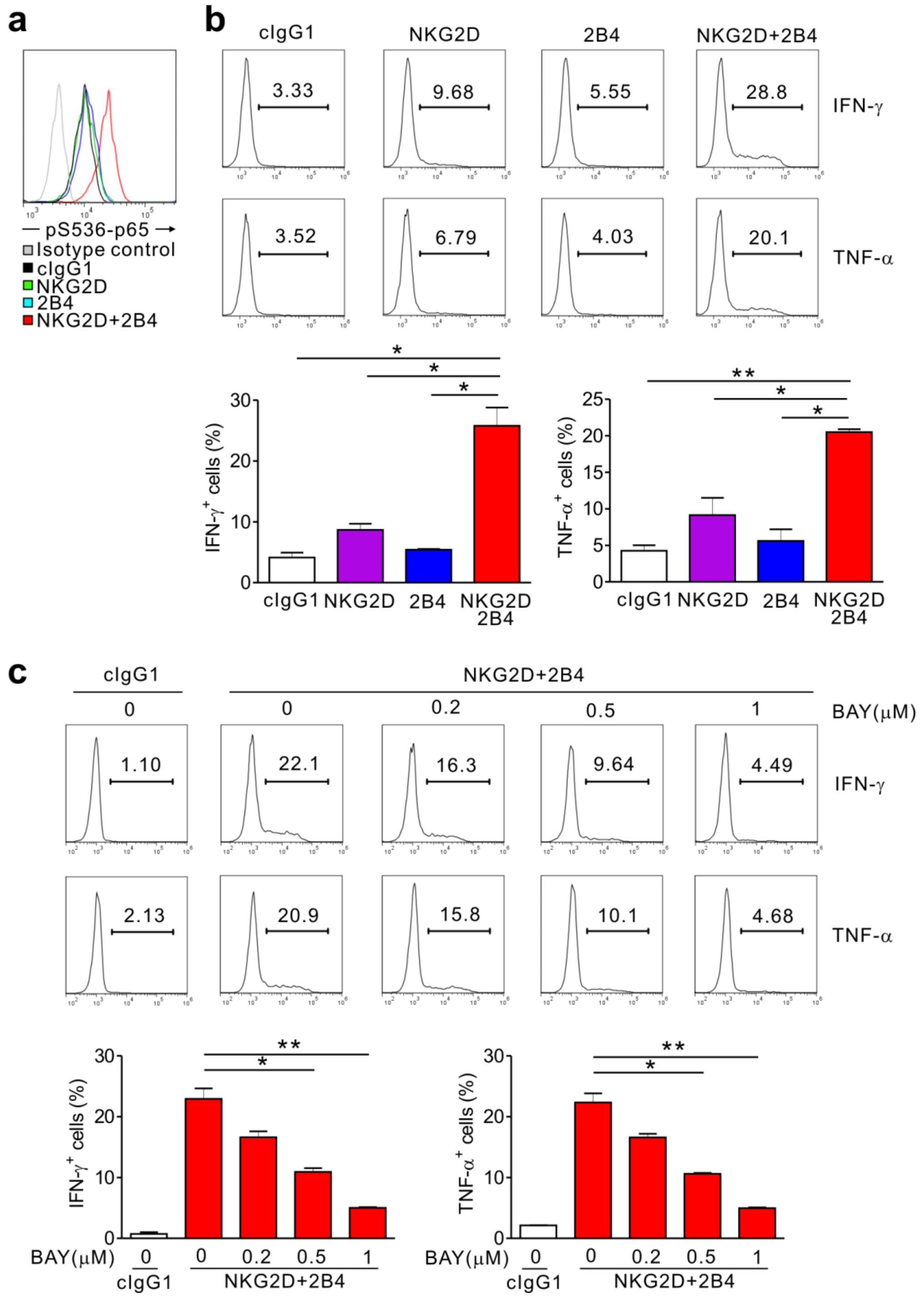

Supplementary figure 5. The synergistic production of cytokines by primary NK cells is NF-κB-dependent.

(**a**) Phosphorylation of NF-κB p65 subunit in primary rested NK cells that were stimulated with NKG2D and/or 2B4 as in Fig. 1**a** for 5 min. Cells were fixed, permeabilized, stained with Alexa

Fluor 488-conjugated isotype control mAb or mAb to phospho-p65 at serine 536, and analyzed by flow cytometry.

(**b**) Frequency of NK cells that displayed IFN-γ or TNF-α expression after stimulation of PBMCs with P815 target cells as indicated. After incubation for 6 h, cells were stained for surface markers with mAbs to CD3 and CD56. Cytokine production by NK cells was measured by intracellular expression of IFN-γ or TNF-α in CD3-CD56+ NK cells. Representative result (top) and statistical bar charts (bottom) are shown. Values represent mean ± s.e.m.

(**c**) Frequency of NK cells that displayed IFN-γ or TNF-α expression after pretreatment of PBMCs with BAY11-7082 at the indicated dose for 1 h and then stimulation with P815 target cells as indicated in the presence of the inhibitor. After incubation for 6 h, cells were stained and analyzed by flow cytometry as in (**b**). Representative result (top) and statistical bar charts (bottom) are shown. Values represent mean ± s.e.m. $*P < 0.05$; $**P < 0.01$ (two-sided Student's *t*-test). Data are representative of at least three independent experiments. Statistical bar charts in panel b and c show pooled data from three experiments.

Supplementary fig. 6

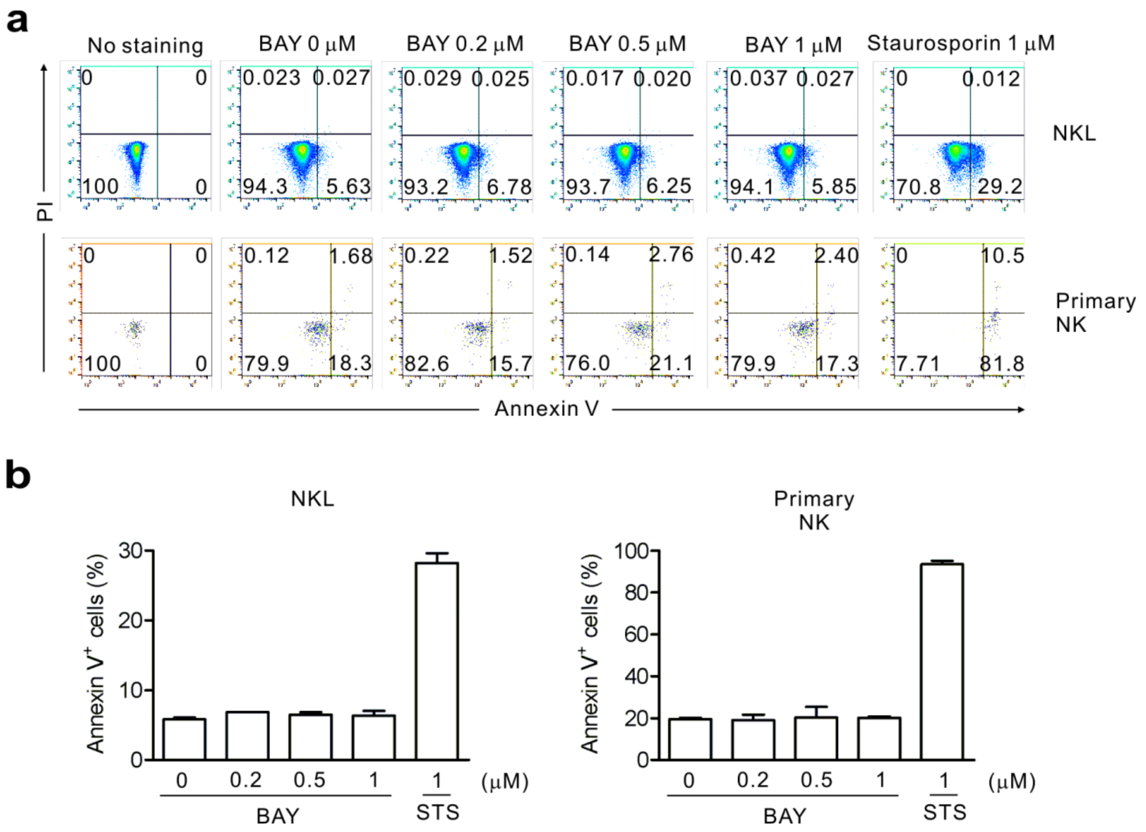

Supplementary figure 6. Effects of BAY11-7082 on the viability of NK cells.

Rested NKL cells or primary rested NK cells after expansion were treated with BAY11-7082 or Staurosporin at the indicated dose for 9 h, then stained with Annexin-V and PI, and analyzed with flow cytometry. Representative result (**a**) and statistical bar charts (**b**) for the frequency of Annexin V+ cells are shown. Values represent mean ± s.d. Data are representative of three independent experiments.

Supplementary fig. 7

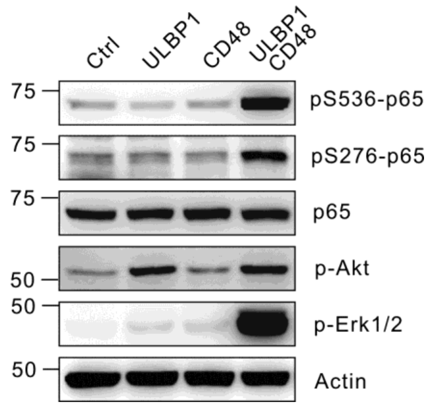

Supplementary figure 7. Synergistic phosphorylation of p65 by NKG2D and 2B4 ligands. Rested NKL cells were stimulated with P815 cells expressing ULBP1 and/or CD48. Cell lysates were immunoblotted with Abs specific for pS536-p65, pS276-p65, p65, p-Akt, p-Erk1/2, or actin. Data are representative of three independent experiments.

Supplementary fig. 8

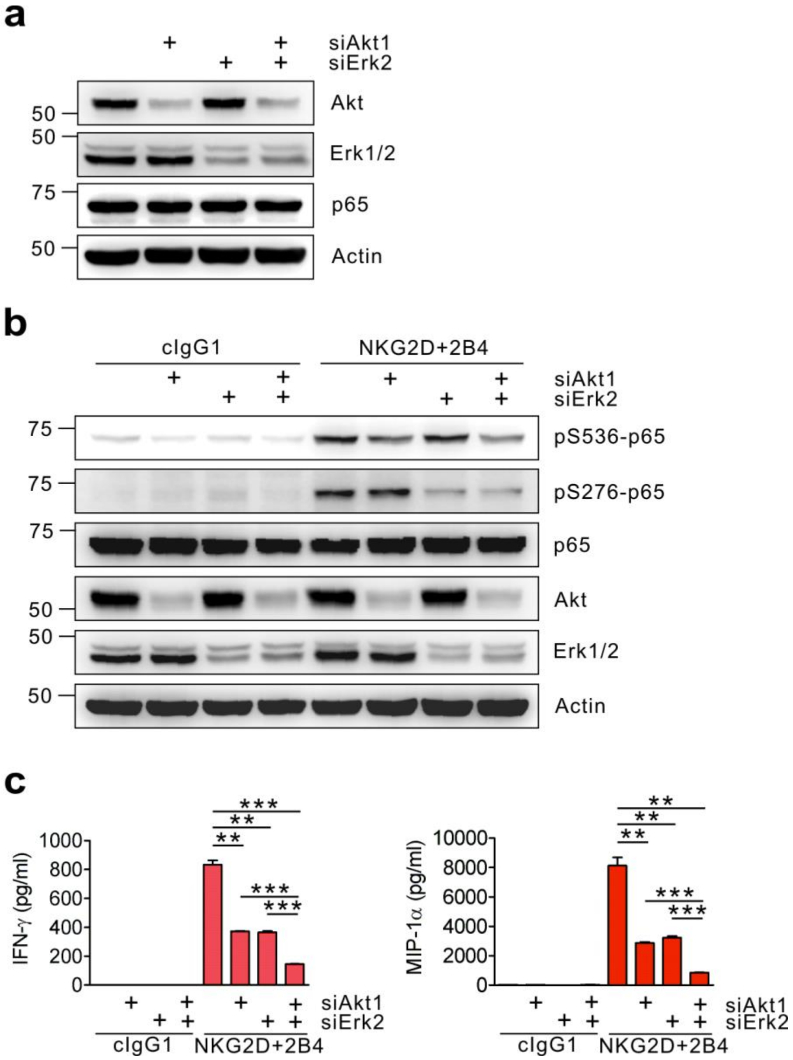

Supplementary figure 8. Requirement of Akt1 and Erk2 for optimal p65 phosphorylation and IFN-γ and MIP-1α production following coactivation.

(**a**) NKL cells were transfected with control siRNA or siRNAs specific for Akt1 and/or Erk2. Lysates were immunoblotted for Akt, Erk1/2, p65, or actin.

(**b**) Rested NKL cells transfected with control siRNA or siRNAs specific for Akt1 and/or Erk2 were stimulated with NKG2D and 2B4 by receptor crosslinking. Lysates were immunoblotted for pS536-p65, pS276-p65, p65, Akt, Erk1/2, or actin.

(**c**) Cytokine release assays with rested NKL cells after transfection with control siRNA or siRNAs specific for Akt1 and/or Erk2 and then stimulation with NKG2D and 2B4 for 8 h. Thereafter, IFN-γ (left) and MIP-1α (right) in the supernatants were measured by ELISA. Values represent mean ± s.d. **P<0.01; ***P<0.001 (two-sided Student's t-test). Data are representative of three independent experiments.

Supplementary fig. 9

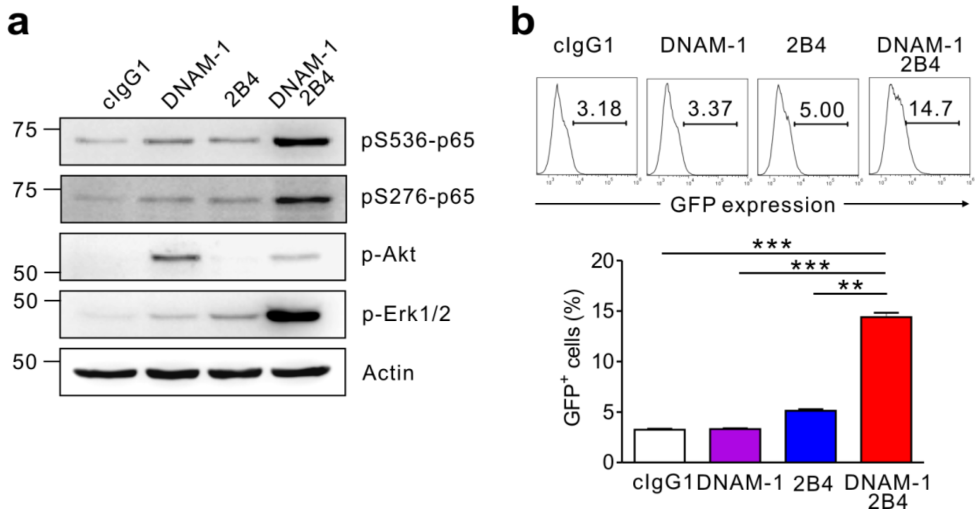

Supplementary figure 9. DNAM-1 synergizes with 2B4 to induce competent p65 phosphorylation and NF-κB activation.

(**a**) Primary rested NK cells after expansion were stimulated with DNAM-1 and/or 2B4 for 2 min. Thereafter, the lysates were immunoblotted with Abs to pS536-p65, pS276-p65, p-Akt, p-Erk1/2, or actin.

(**b**) Rested NKL-κB-GFP cells were stimulated with plate-immobilized mAbs specific for DNAM-1 and/or 2B4 for 6 h. GFP expression in NKL-κB-GFP cells was analyzed by flow cytometry, and representative result (top) and statistical bar charts (bottom) are shown. Values represent mean ± s.d. ***P* < 0.01; ****P* < 0.001 (two-sided Student's *t*-test). Data are representative of three independent experiments.

Supplementary fig. 10

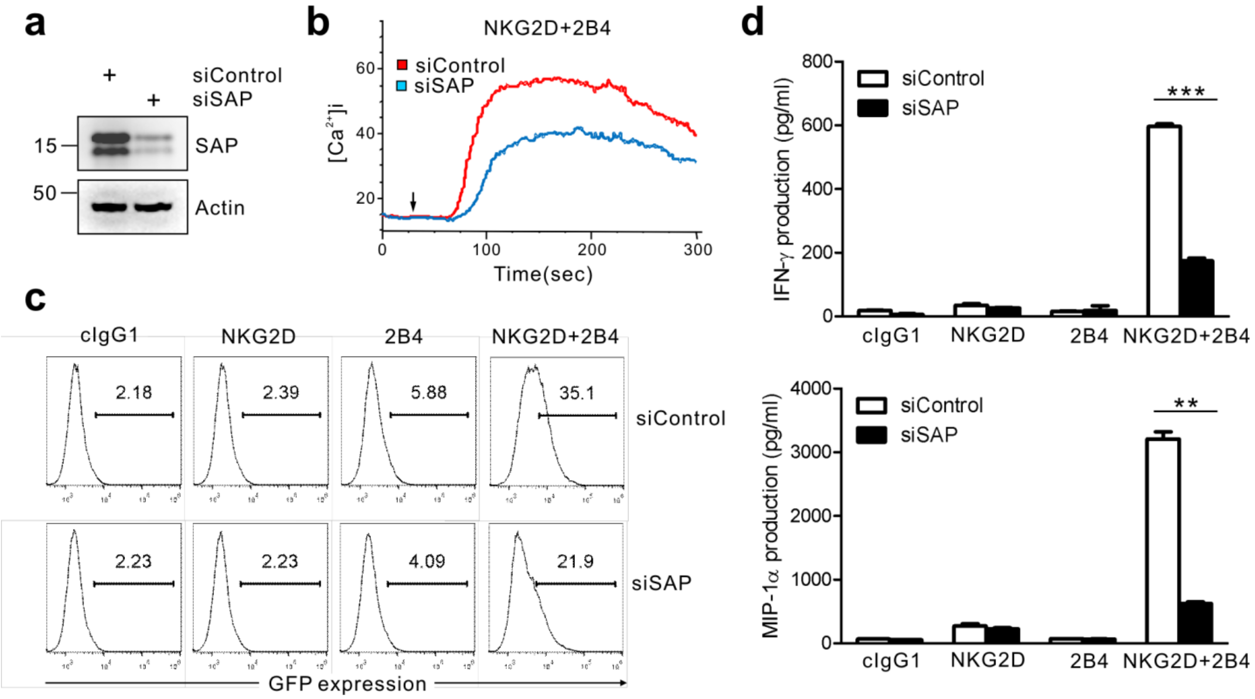

Supplementary figure 10. SAP is required for NF-κB activation by NKG2D and 2B4 coactivation.

(**a**) NKL cells were transfected with 300 pmoles of control siRNA or siRNA specific for SAP. After 24 h, the cells were rested for another 24 h, and the lysates were immunoblotted for SAP or actin.

(**b**) Ca^{2+} mobilization in rested NKL cells transfected with control siRNA or SAP-specific siRNA. NKL cells were stimulated through NKG2D and 2B4 (indicated by the arrow) after the measurement of baseline Ca^{2+} concentrations for 30 s. Changes in fluorescence are shown as a function of time.

(**c**) Rested NKL-κB-GFP cells transfected with control siRNA or SAP-specific siRNA were stimulated with plate-immobilized mAbs specific for NKG2D and/or 2B4 for 6 h. GFP expression in the reporter NKL cells was analyzed using flow cytometry.

(**d**) Cytokine release assays with rested NKL cells transfected with control siRNA or SAP-specific siRNA and stimulated with NKG2D and/or 2B4. After incubation for 12 h, IFN-γ (top) and MIP-1α (bottom) in the supernatants were measured by ELISA. Values represent the mean ± s.d. $^{**}P < 0.01$; $^{***}P < 0.001$ (two-sided Student's *t*-test). Data are representative of three independent experiments.

Supplementary fig. 11

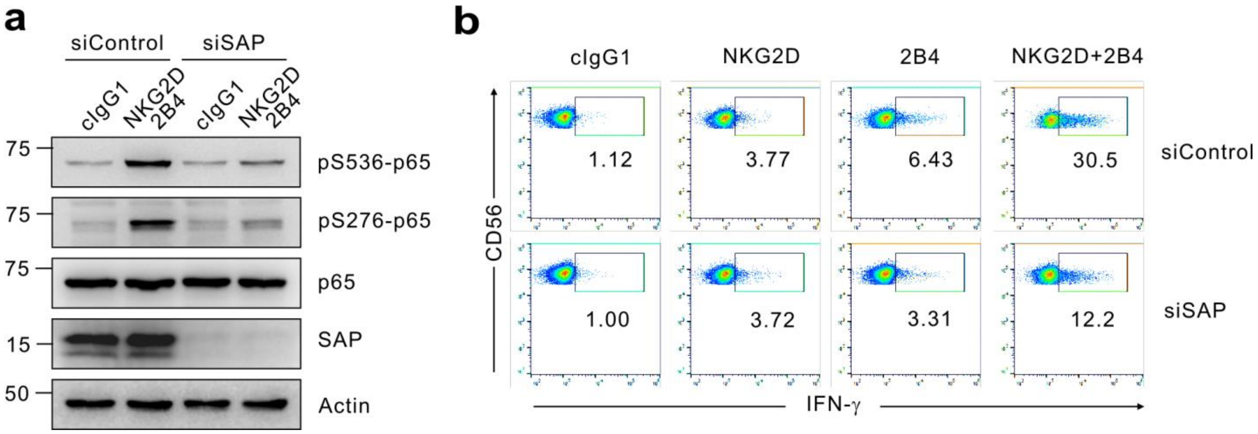

Supplementary figure 11. SAP is required for p65 phosphorylation and IFN-γ expression in primary NK cells via NKG2D and 2B4 coactivation.

(**a**) Primary rested NK cells after expansion that were transfected with control siRNA or SAP-specific siRNA were stimulated with NKG2D and 2B4 by receptor crosslinking. Lysates were immunoblotted for pS536-p65, pS276-p65, p65, SAP, or actin.

(**b**) Primary rested NK cells after expansion that were transfected with control siRNA or SAP-specific siRNA were stimulated with P815 target cells as indicated. After incubation for 6 h, cells were stained, and frequency of NK cells that displayed intracellular IFN-γ expression was analyzed by flow cytometry. Data are representative of three independent experiments.

Supplementary fig. 12

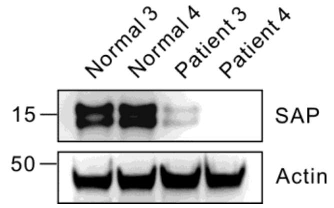

Supplementary figure 12. SAP deficiency in XLP1 NK cells.

Total lysates of primary expanded NK cells from normal (normal 3 and 4) or XLP1 patient (patient 3 and 4) donors were immunoblotted for SAP and actin.

Supplementary fig. 13

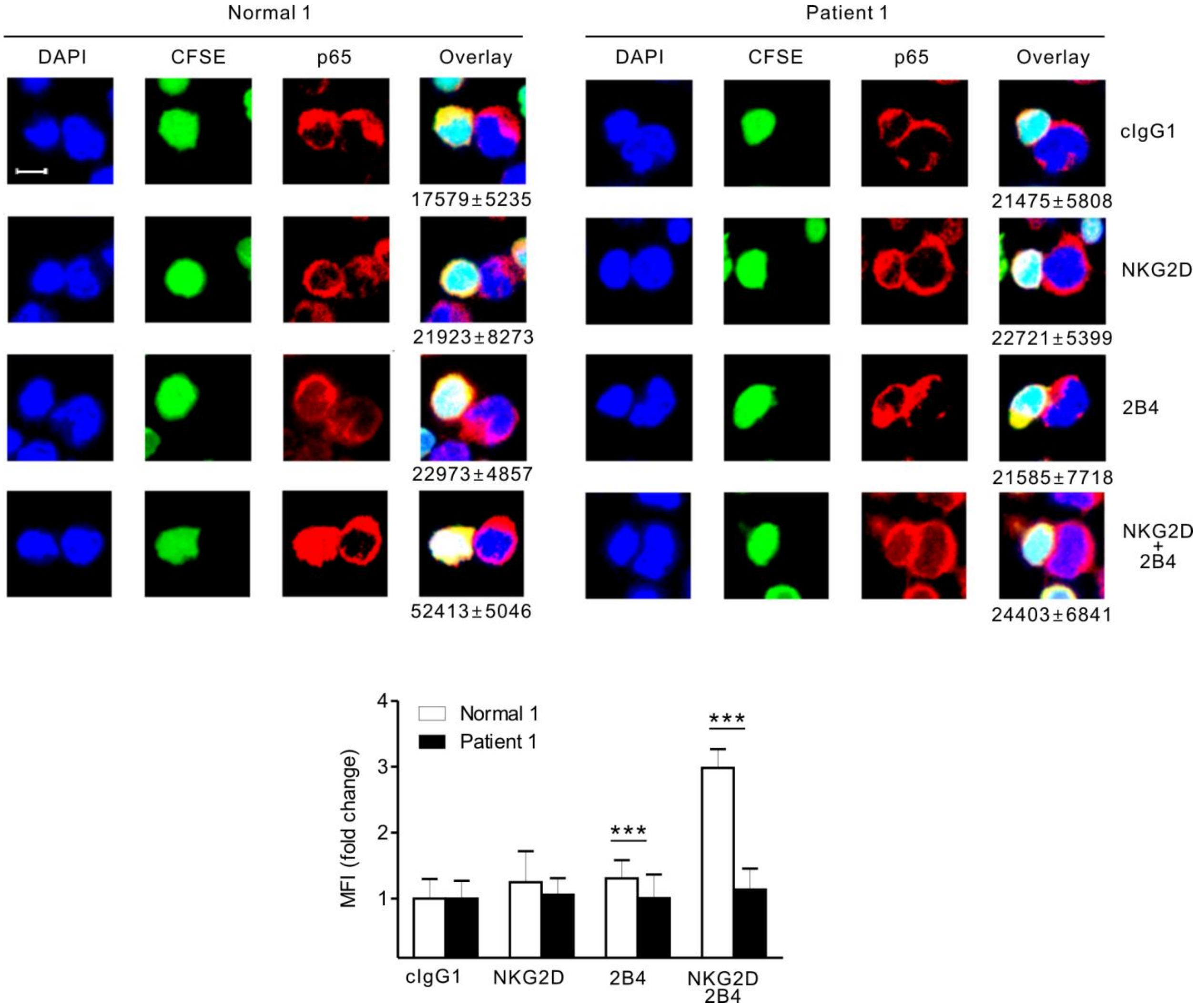

Supplementary figure 13. XLP1 NK cells have defects in p65 nuclear translocation in response to NKG2D and 2B4 coactivation.

Representative confocal images (top) of conjugates between CFSE (green)-loaded primary rested NK cells from normal or XLP1 donor and P815 target cells as indicated. Conjugates were fixed, permeabilized, and stained with DAPI (blue) and mAb to p65, anti-mouse IgG-Biotin followed with Alexa Fluor 647 (red)-Streptavidin. Mean nuclear MFI ± s.d. of p65 in ≥ 30 NK-target cell conjugates are shown beneath the overlay image. Statistical bar charts (bottom) for MFI of p65 in the nucleus are represented as fold change. Values represent mean ± s.d. ***P*<0.001 (two-sided Student's *t*-test). Scale bar, 5 μm.

Supplementary fig. 14

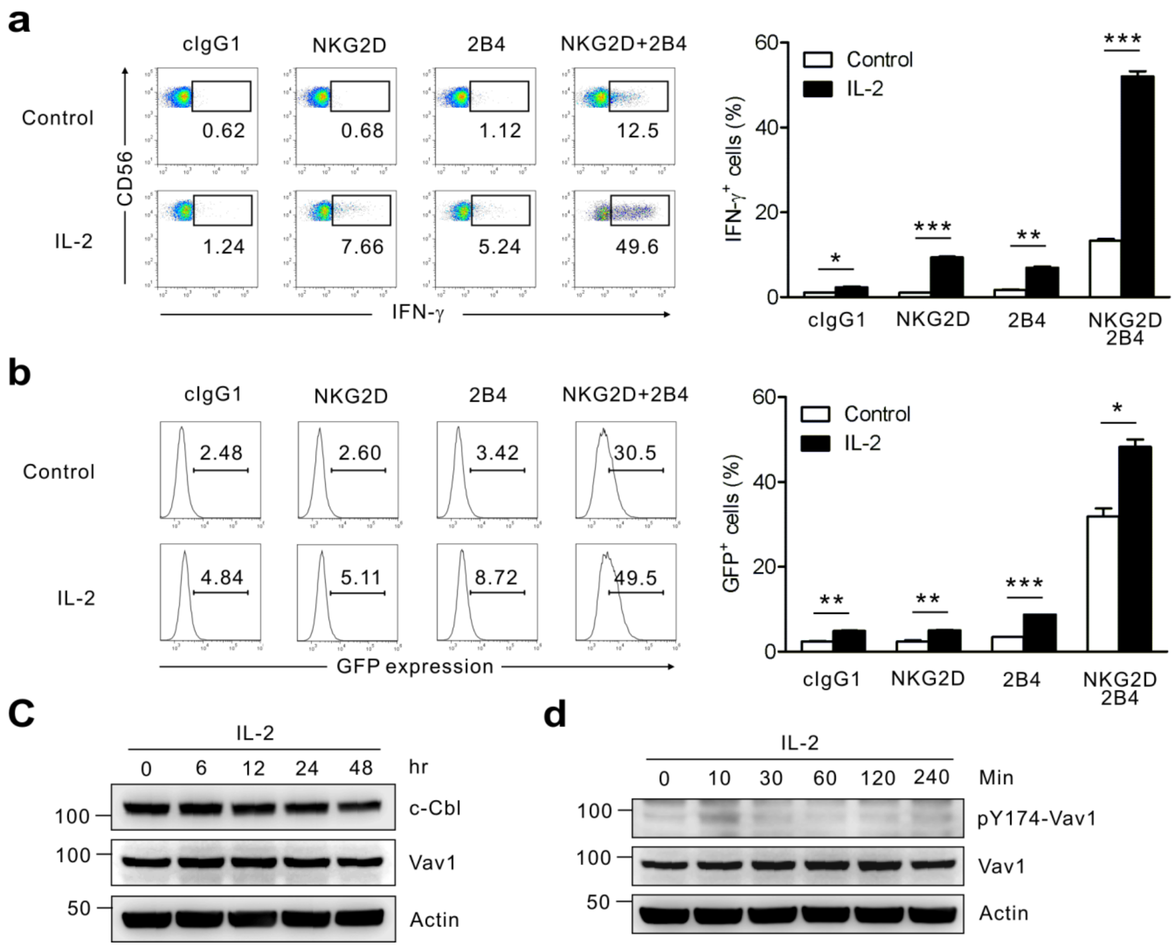

Supplementary figure 14. Preactivation with IL-2 enhances the responsiveness of NK cells for IFN-γ expression and NF-κB activation.

(**a**) Frequency of NK cells that displayed IFN-γ expression after pretreatment of PBMCs with or without IL-2 (400 U/ml) and then stimulation with P815 target cells as indicated. After incubation for 6 h, cells were stained and analyzed by flow cytometry. Representative result (left) and statistical bar charts (right) are shown. Values represent mean ± s.d.

(**b**) NKL-κB-GFP cells were rested or pretreated with IL-2 (400 U/ml) for 20 h and then stimulated with plate-immobilized mAbs specific for NKG2D and/or 2B4 for 6 h in the absence or presence of IL-2 (400 U/ml). GFP expression in NKL-κB-GFP cells was analyzed by flow cytometry, and representative result (left) and statistical bar charts (right) are shown. Values represent mean ± s.d.

(**c** and **d**) Rested NKL cells were stimulated with IL-2 (400 U/ml) for the indicated time. Cell lysates were immunoblotted for c-Cbl, Vav1, or actin (**c**) or phospho-Vav1 at tyrosine 174 (pY174), Vav1, or actin (**d**). *P<0.05; **P<0.01; ***P<0.001 (two-sided Student's *t*-test). Data are representative of three independent experiments.

Supplementary fig. 15

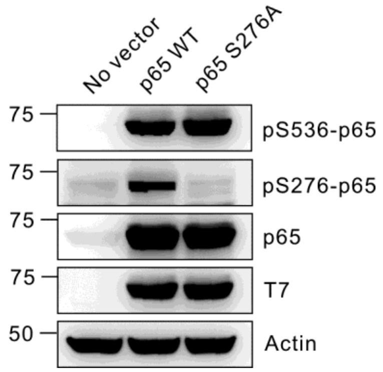

Supplementary figure 15. The specificity of the antibody against p65 at serine 276.
293T cells were transfected with pEV3s-T7-RelA or pEV3s-T7-RelA S276A expression vector. After 48 h incubation, cell lysates were immunoblotted for phospho-p65 at serine 536 (pS536), phospho-p65 at serine 276 (pS276), p65, T7, or actin. Data are representative of three independent experiments.

Supplementary figure 16. Full-length immunoblots.

Followings are the full-length immunoblots for Figure 1(a,b,d), Figure 2b, Figure 3 (a,b,d,e), Figure 4a, Figure 5(a,d), Figure 6d, Figure 7(a,c), Figure S7, Figure S8(a,b), Figure S9a, Figure S10a, Figure S11a, Figure S12, Figure S14(c,d), and Figure S15.

Figure 1

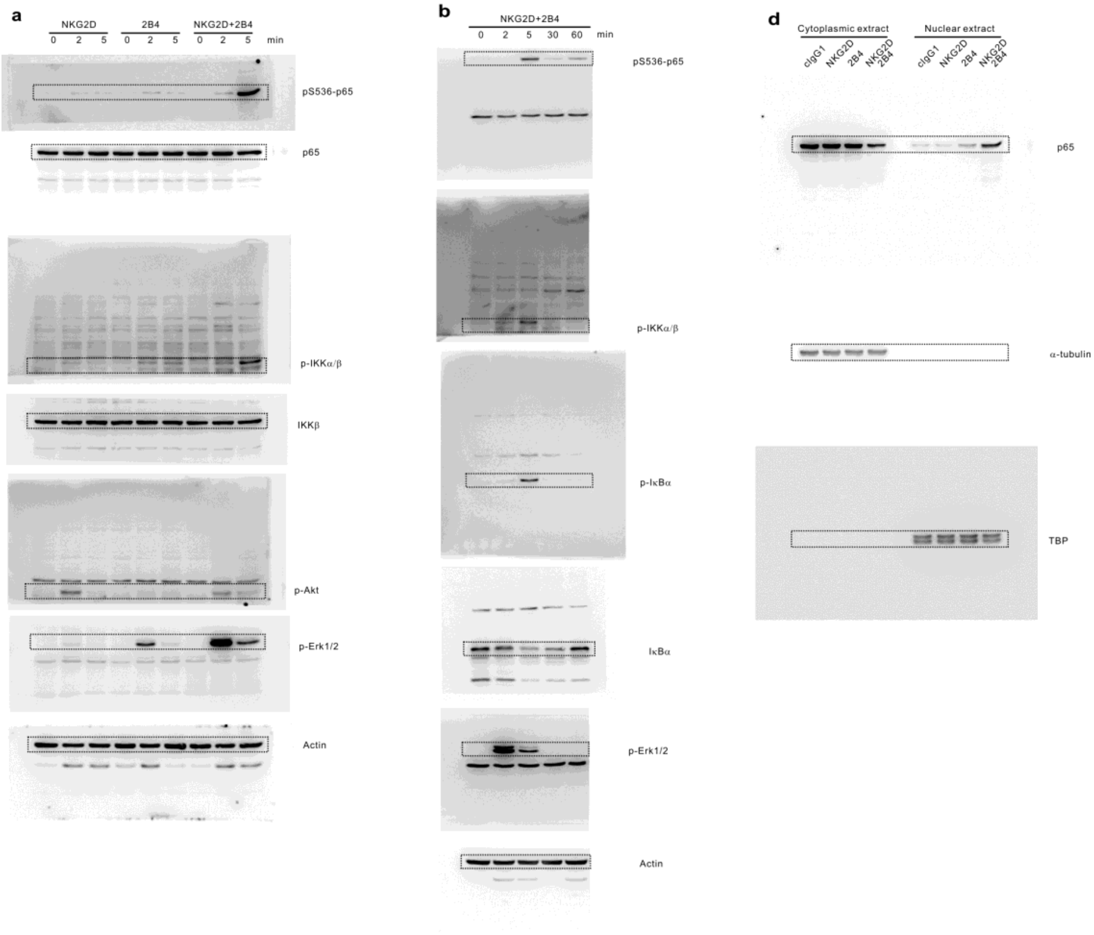

Figure 2

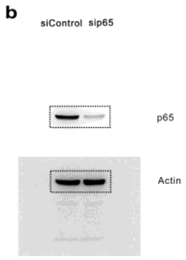

Figure 3

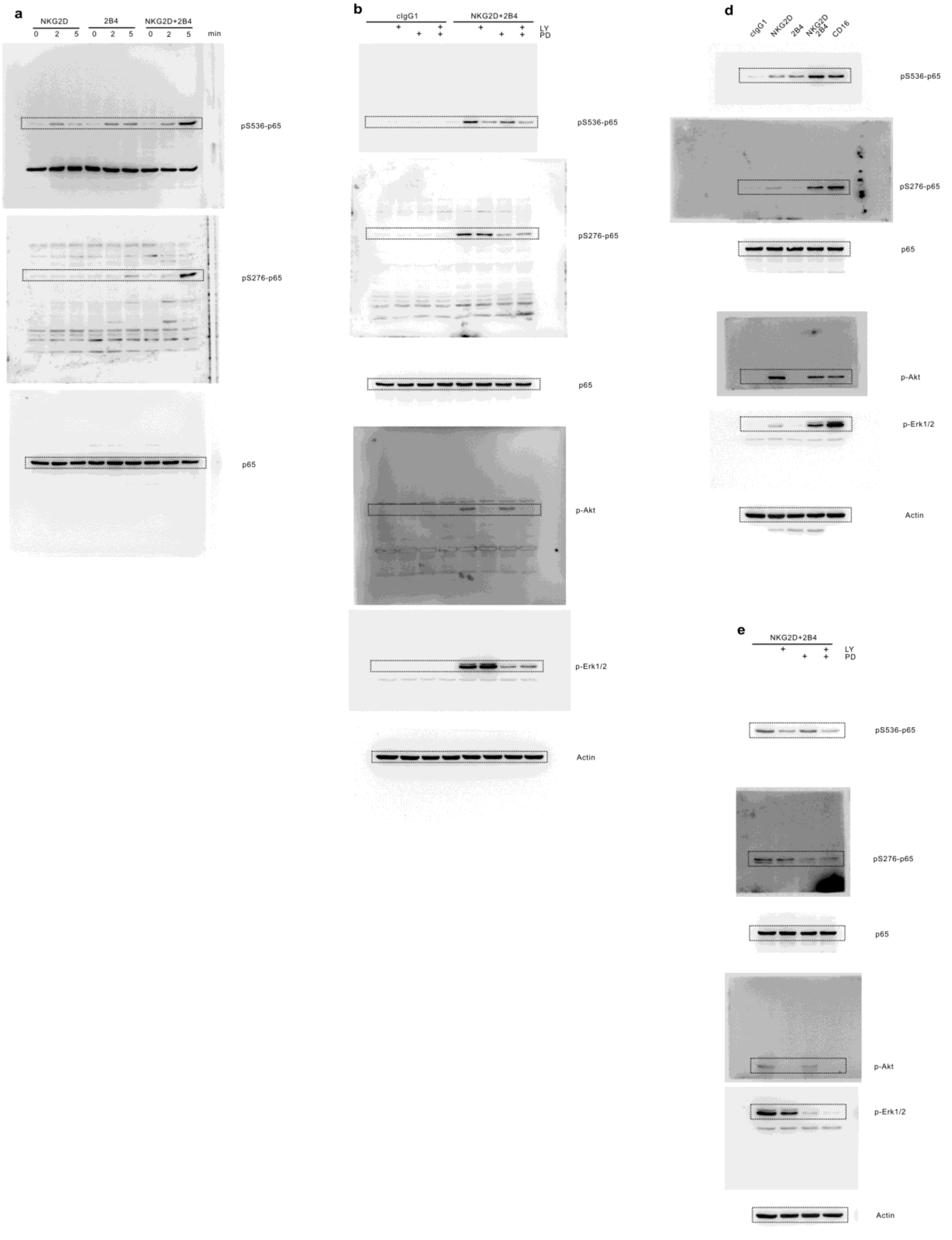
a
NKG2D
2B4
NKG2D+2B4
0 2 5 0 2 5 0 2 5 min
pS536-p65
pS276-p65
p65
b
cIgG1
NKG2D+2B4
LY
PD
pS536-p65
pS276-p65
p65
p-Akt
p-Erk1/2
Actin
d
cIgG1
NKG2D
2B4
NKG2D 2B4
CD16
pS536-p65
pS276-p65
p65
p-Akt
p-Erk1/2
Actin
e
NKG2D+2B4
LY
PD
pS536-p65
pS276-p65
p65
p-Akt
p-Erk1/2
Actin

Figure 4

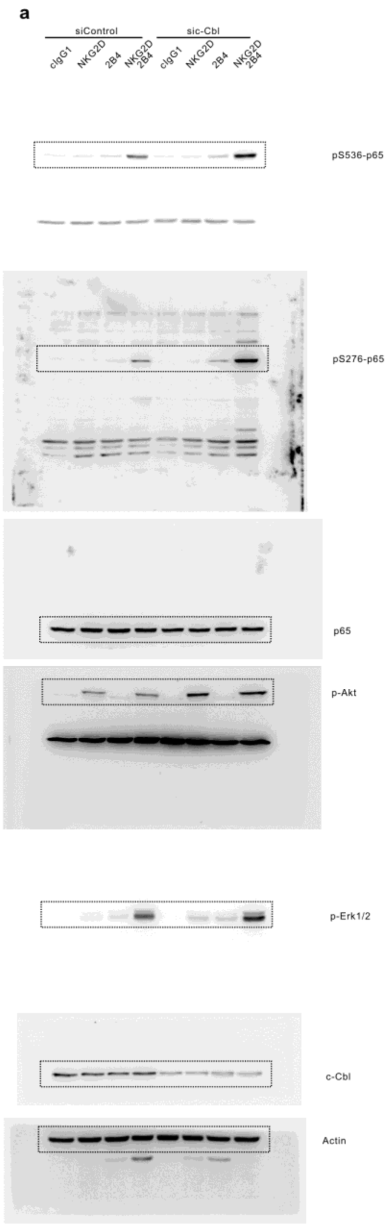

Figure 5

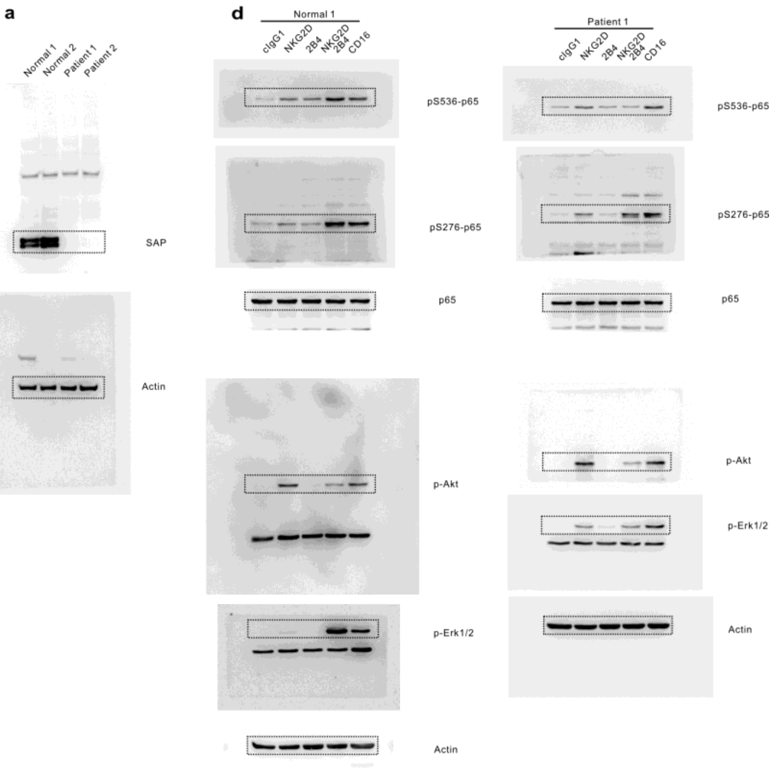

Figure 6

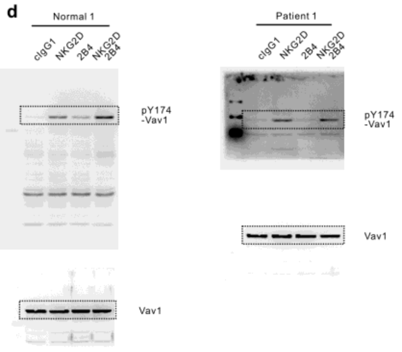

Figure 7

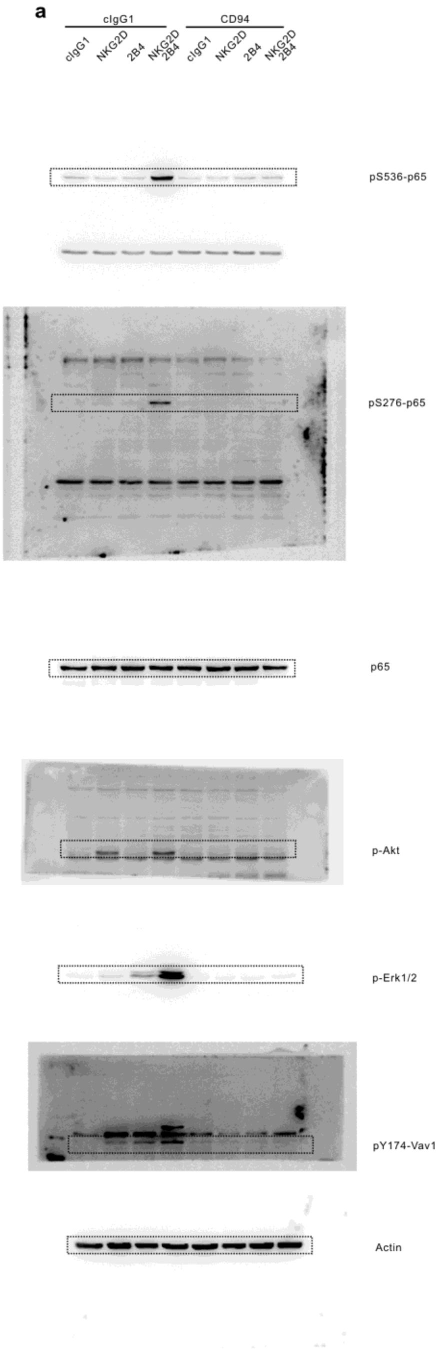
a
cIgG1
CD94
cIgG1
NKG2D
2B4
NKG2D 2B4
cIgG1
NKG2D
2B4
NKG2D 2B4
pS536-p65
pS276-p65
p65
p-Akt
p-Erk1/2
pY174-Vav1
Actin

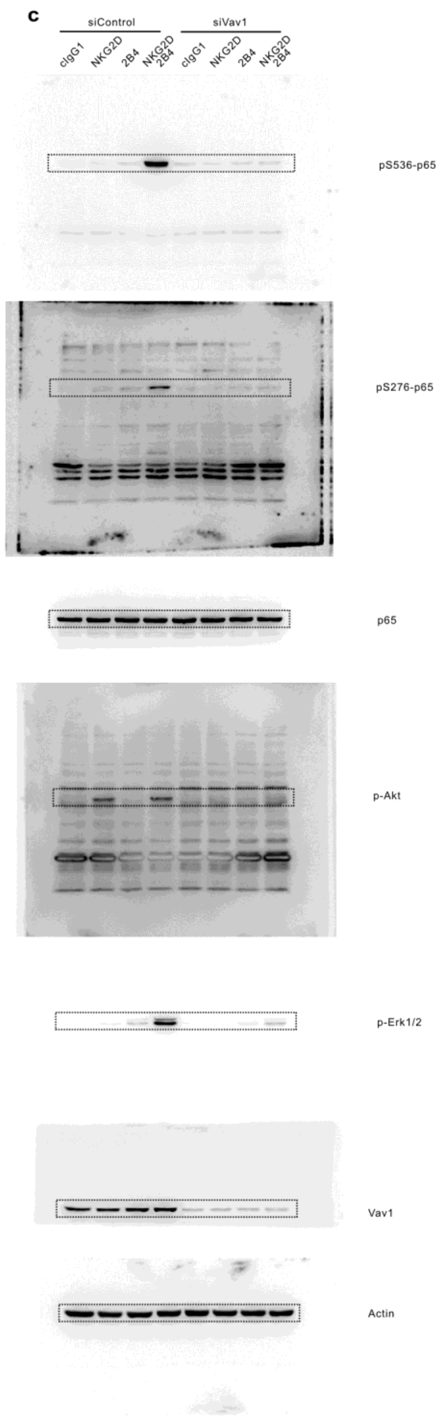
c
siControl
siVav1
cIgG1
NKG2D
2B4
NKG2D 2B4
cIgG1
NKG2D
2B4
NKG2D 2B4
pS536-p65
pS276-p65
p65
p-Akt
p-Erk1/2
Vav1
Actin

Figure S7

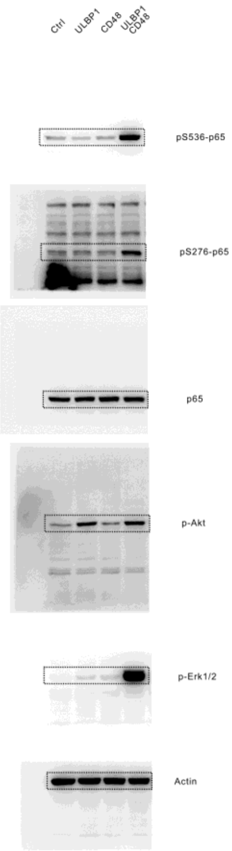

Figure S8

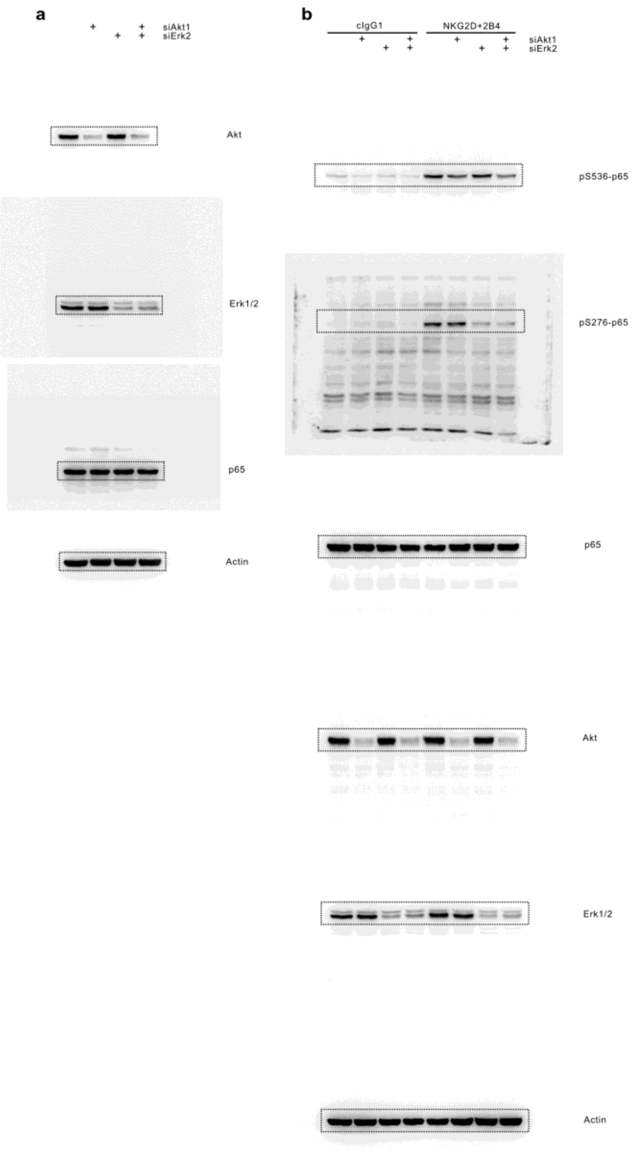

Figure S9

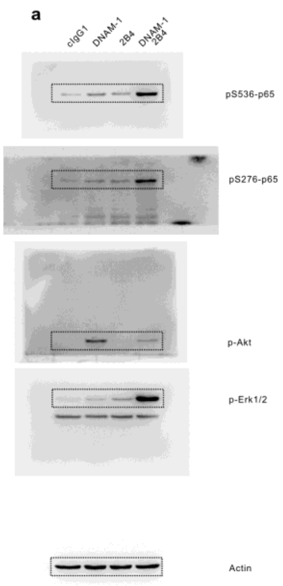

Figure S10

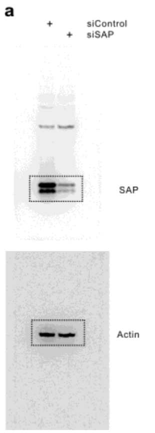

Figure S11

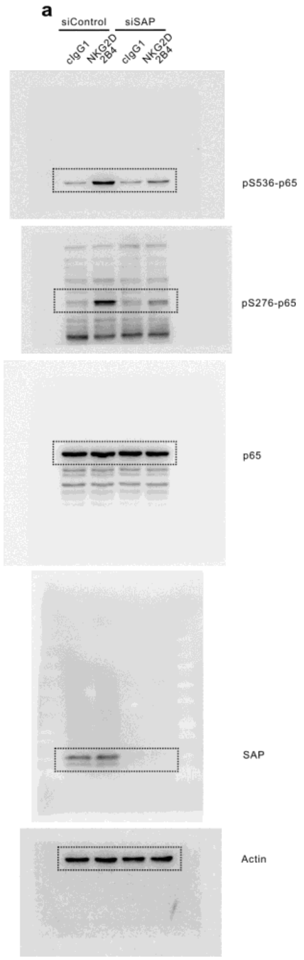

Figure S14

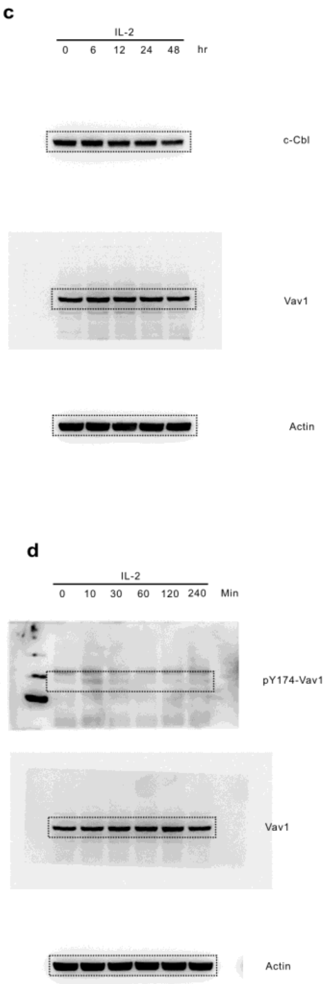

Figure S15

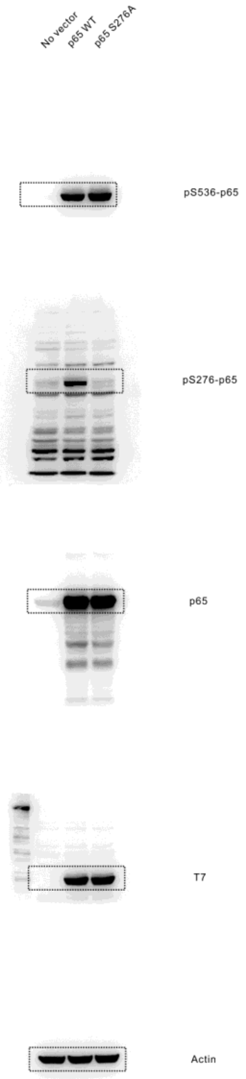

Figure S12

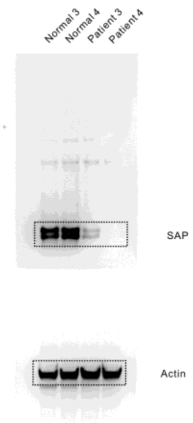

Supplementary Table 1. List of primers used for real-time PCR analyses of the indicated genes

Functional category	Protein (*Gene*)	Primer (5'-3')
Cytokine	IFN-γ (*IFNG*)	Forward: GCATCGTTTTGGGTTCTCTTGGCTGTTACTGC<br>Reverse: CTCCTTTTTCGCTTCCCTGTTTTAGCTGCTGG
	TNF-α (*TNF*)	CAGAGGGCCTGTACCTCATC<br>GGTTGAGGGTGTCTGAAGGA
Chemokine	MIP-1α (*CCL3*)	GCTGACTACTTTGAGACGAGC<br>CCAGTCCATAGAAGAGGTAGC
	MIP-1β (*CCL4*)	CCAAACCAAAAGAAGCAAGC<br>AGAAACAGTGACAGTGGACC
	RANTES (*CCL5*)	ATCCTCATTGCTACTGCCCTC<br>GCCACTGGTGTAGAAATACTCC
Cytolytic pathway	Granzyme B (*GZMB*)	CCCTGGGAAAACACTCACACA<br>CACAACTCAATGGTACTGTCGT
	Perforin (*PRF1*)	CGCCTACCTCAGGCTTATCTC<br>CCTCGACAGTCAGGCAGTC
Death receptor apoptotic pathway	FASL (*FASLG*)	CTTGGTAGGATTGGGCCTGG<br>TGTGTGCATCTGGCTGGTAG
	TRAIL (*TNFSF10*)	CCGGCTGCCTGGCTGACTTAC<br>TCAGCACGCAGGTCTGTCCC
NF-κB pathway	IκBα (*NFKBIA*)	TGGCCTTCCTCAACTTCCAGAACA<br>CTCAGCAATTTCTGGCTGGTTGGT
	p65 (*RELA*)	GAAGAAGAGTCCTTTCAGCG<br>GGGAGGACGTAAAGGGATAG
	BCL10 (*BCL10*)	CCCGCTCCGCCTCCTCTCCTT<br>GGCGCTTCTTCCGGGTCCG
Apoptosis	BCL-XL (*BCL2L1*)	GACTGAATCGGAGATGGAGACC<br>GCAGTTCAAACTCGTCGCCT
	MCL1 (*MCL1*)	CATTCCTGATGCCACCTTCT<br>TCGTAAGGACAAAACGGGAC
IL-2 receptor	CD122 (*IL2RB*)	TCATCATCTTAGTGTACTTGCTGATCA<br>GGTGTTACACTTCAGGACCTTCTTC
	CD132 (*IL2RG*)	GGAGCAATACTTCAAAAGAGAATCCT<br>CCCATGGAGCCAACAGAGAT
Cytolytic granule exocytosis	Munc13-4 (*UNC13D*)	GCCAGGCCATCAAGATAAGG<br>CTCGGGGGAGAAGTGGTG
	Rab27a (*RAB27A*)	AGCAGGGCAGGAGAGGTTTCGTA<br>TGCTATGGCTTCCTCCTCTTTCAC
	Syntaxin11 (*STX11*)	ACAGGTTTCCTTCTCCATCG<br>TGCTGGTCATATTGCTTGGA
House keeping gene	β-actin (*ACTB*)	ACTCCATCATGAAGTGTGACG<br>CATACTCCTGCTTGCTGATCC
